# Host response signatures across sepsis aetiologies in India: a single centre observational study

**DOI:** 10.1016/j.lansea.2026.100752

**Published:** 2026-03-21

**Authors:** Jason J. Biemond, Venkat A. Earny, Harjeet Virk, Anusree Adukkadukkam, Anjali Girish, Augustijn M. Klarenbeek, Hessel Peters-Sengers, Joe M. Butler, Chiranjay Mukhopadhyay, W. Joost Wiersinga, Tom van der Poll

**Affiliations:** a Centre for Infection and Molecular Medicine, Amsterdam University Medical Centre, Location AMC, University of Amsterdam, Amsterdam, the Netherlands; b Department of Microbiology, Kasturba Medical College, Manipal Academy of Higher Education, Manipal, India; c Centre for Emerging and Tropical Diseases (CETD), Manipal Academy of Higher Education, Manipal, India; d Department of Microbiology, Queen Alexandra Hospital, Portsmouth Hospitals University National Health Service Trust, Portsmouth, United Kingdom; e Manipal Institute of Virology, Manipal Academy of Higher Education, Manipal, India; f Department of Epidemiology and Data Science, Amsterdam University Medical Centre, Location Vrije Universiteit Amsterdam, Amsterdam, the Netherlands; g Amsterdam Institute for Immunology and Infectious Diseases, Amsterdam University Medical Centre, Amsterdam, the Netherlands; h Division of Infectious Diseases, Department of Medicine, Amsterdam University Medical Centre, University of Amsterdam, Amsterdam, the Netherlands

**Keywords:** Sepsis, India, Tropical infection, ICU, Host response

## Abstract

**Background:**

Characterising the host response in sepsis is essential to understand its biological heterogeneity and to inform more precise diagnostic and therapeutic strategies. Existing evidence on sepsis comes predominantly from studies conducted in high-income countries (HICs), despite the highest burden in low- and middle-income countries (LMICs). We aimed to address this gap by identifying shared and pathogen-specific host response patterns among different infectious causes of sepsis in a tertiary care centre in India.

**Methods:**

Patients fulfilling sepsis-3 criteria were enrolled within 24 h of intensive care unit admission in a tertiary care centre in Manipal, Karnataka, India. We measured 27 plasma biomarkers reflecting key pathophysiological domains (endothelial activation and coagulation, organ damage and inflammation, cytokine response, and chemokine release) to delineate host response profiles across sepsis aetiologies.

**Findings:**

We included 956 sepsis patients, and a causative pathogen was identified in 54·1% (338 bacterial, 146 viral, 33 polymicrobial). The causative pathogen explained a significant proportion of biomarker variation (34·5% of explained variance; 9·4% of all variances). While bacterial sepsis was associated with stronger host response changes across all domains when compared to viral sepsis, notable variation was found within these microbial categories. *Orientia tsutsugamushi* (causing scrub typhus) triggered the most profound host response alterations among bacterial causes, whereas viral haemorrhagic fever (dengue, Kyasanur forest disease) causing pathogens induced higher biomarker levels across all pathophysiological domains compared to SARS-CoV-2 or influenza within viral sepsis.

**Interpretation:**

These findings highlight the biological heterogeneity of sepsis and the complexity of host–pathogen interactions in a setting with a diverse range of causative organisms.

**Funding:**

10.13039/501100000780European Union, 10.13039/100019573Amsterdam UMC, and 10.13039/100019305Manipal Academy of Higher Education.

**Research in context**

**Evidence before this study**

On February 3, 2026 we conducted a PubMed search to identify studies reporting biomarker signatures across sepsis aetiologies in adult ICU patients from India using (search) keywords for Title/Abstract (India AND sepsis AND aetiology AND ICU AND biomarker). A total of 23 articles were identified. Six articles were reviews discussing various sepsis complications. Five articles were prognostic studies in children. Three articles reported different biomarker expressions in patients with and without sepsis. Four articles used biomarkers to predict mortality in patients with sepsis. There were no articles that described differences in biomarker profiles depending on the causative pathogen on the ICU in India.

**Added value of this study**

We conducted a large multi-pathogen analysis of the host response in 956 intensive care unit patients with sepsis in a tertiary care centre in India, a country that contributes more than 25% of global sepsis related deaths. In over half of patients, a causative pathogen was identified. By measuring 27 plasma biomarkers reflective of pathophysiological domains implicated in sepsis (endothelial activation and coagulation, organ damage and inflammation, cytokine response, and chemokine release), we characterised host-response patterns across microbial aetiologies. The infecting pathogen explained a substantial proportion of biomarker variation. Bacterial sepsis produced stronger systemic changes than viral sepsis overall, yet notable heterogeneity was observed within these groups. Scrub typhus (caused by *Orientia tsutsugamushi*) induced a highly distinct response among bacterial infections. Within viral infections, haemorrhagic fevers (dengue and Kyasanur forest disease) caused stronger activation of multiple domains when compared to respiratory viruses (influenza and SARS-CoV-2). This analysis provides a comprehensive host response data in sepsis caused by Kyasanur forest disease. By combining broad biomarker panels with diverse pathogens, this study highlights heterogeneity in the biology of sepsis, particularly in a geographic region relevant for the global burden, that would otherwise not be apparent from single-domain or single-pathogen analyses.

**Implications of all the available evidence**

These data emphasise that results from HIC cohorts cannot be directly applied to LMIC populations with different pathogen landscapes. Recognition of pathogen-specific host-response patterns is critical for advancing precision medicine in sepsis, clinical trials may need pathogen-stratified designs, and biomarker-guided approaches must be validated in settings with endemic infections. The current results are also important from a global health equity perspective, as the populations most affected by sepsis have been least represented in biological studies.

## Introduction

Sepsis is a critical and complex condition characterised by a dysregulated host response to infection, associated with systemic inflammation, organ dysfunction, and a high mortality.^1^ It is a heterogeneous syndrome that manifests differently depending on the pathogen involved, age, sex, premorbid conditions, environmental factors and the host immune response.^2^ While significant research on sepsis and host responses has been conducted in high-income countries (HICs), there is a notable lack of data from low- and middle-income countries (LMICs), where the burden of sepsis is disproportionately high.^3^ The pathogens that contribute to sepsis in LMICs are often distinct, with many being endemic to these regions.^4^ Moreover, patient characteristics and ecological factors differ for LMICs which may influence host responses to infection.

We recently reported on the aetiology and outcome of community-acquired sepsis in India,^4^ a country that contributes 26·4% to global sepsis-related deaths.^3^ This study highlighted the burden of tropical infections causing sepsis and the inordinate impact on younger individuals and those with a lower socioeconomic background.^4^ Despite the high burden and distinct pathogen spectrum, comprehensive characterisation of the sepsis host response in India remains scarce. Existing studies have largely evaluated single biomarkers or focused on mortality associations, providing limited insight into the multidomain dysregulation or pathogen-specific response patterns.^5^ It is also not clear on whether the host response signatures described in studies from HICs are generalisable to other regions. Detailed profiling of the host response will be critical to inform context-specific diagnostics, antimicrobial stewardship, and precision-medicine strategies. The current study sought to address the knowledge gap regarding the host response to sepsis in a LMIC belonging to a tropical environment. To this end, we measured 27 biomarkers reflecting changes in key host response pathways implicated in sepsis pathophysiology in a large cohort of patients from a tertiary care centre in India. By comparing the immune signatures associated with different infectious agents, this research aimed to enhance our understanding of pathogen-specific host responses in an LMIC context. We hypothesised that different pathogen classes would be associated with distinct yet partially overlapping host-response signatures across inflammatory, endothelial, and immune-regulatory domains.

## Methods

Patients were recruited as part of the MARS–India study (clinicaltrials.gov identifier NCT03727243), a prospective longitudinal observational study undertaken at a tertiary-care academic hospital in Manipal, Karnataka, India. Trained research personnel screened patients >18 years admitted to the intensive care unit (ICU) between December 2018 and November 2022. For inclusion, patients were identified based on the Sepsis-3 criteria.^1^ Specifically, individuals suspected of infection within 24 h of admission to the ICU, accompanied by an increased Sequential Organ Failure Assessment (SOFA) score of ≥2, were categorised as having sepsis. Non-infected ICU patients were included as critically ill controls. Shock was defined as a serum lactate of >2 mmol/L and vasopressor requirement to maintain haemodynamic stability.^1^ Exclusion criteria included pregnant/breastfeeding women, “withdrawal of care” decision at enrolment, patients with an anticipated duration of hospitalisation in the ICU of <48 h, extra-corporeal circulation in the month preceding inclusion in the case of cardiac surgery, patients with restricted liberty or under legal protection, expected lifespan <3 months due to pre-existing comorbidities, >4 units of blood transfusions in the past week, inability to consent by the patient or next of kin, previous enrolment, or transfer from another hospital ICU (stay >24 h) or ward (stay >72 h). Details on the source of infection, causative microorganisms and antimicrobial resistance patterns of this study population were reported previously.^4^ Clinical data were collected at baseline and throughout the hospital stay. Information included patient demographics (sex, age), comorbidities, medication use, prior hospital admissions, interventions at admission and during hospitalisation, ICU-administered medications, radiology and microbiology results, and all laboratory values obtained during the hospital stay. Patients were followed up at 3 and 6 months after ICU discharge when possible.

Causative microorganisms were classified based on all microbiology results.^4^ Diagnosis and identification of the causative pathogen were determined based on the opinion of the attending physician in the discharge letter and an investigator. With this approach we ensured that only pathogens considered clinically relevant and consistent with the infection episode were classified as causative, eliminating colonising and contaminating microorganisms. In cases where the investigator encountered uncertainty after evaluating the clinical, laboratory, and radiological data, consensus was reached between clinical infectious disease and microbiology study physicians (J. J. B., H. S. V., W. J. W., and C. M.). In 89·6% of cases pathogen assignment by the treating physician was confirmed. Details on which diagnostic test were applied per pathogen are provided in the Appendix, Table S1.

Blood samples for biomarker analysis were collected in EDTA tubes within 24 h of ICU admission. Samples were centrifuged, and plasma was aliquoted and stored at −80 °C within 2 h of collection. 23 host response biomarkers were measured by Luminex using Magpix (R&D, USA), a benchtop multiplexing instrument using xMAP magnetic bead technology for simultaneous detection of multiple analytes. We used a custom/made multiplex assay, which was composed using the “Luminex Assay Customisation Tool & Panel Builder” on R&D's website. Measurements below the limit of quantification were imputed as the lowest extrapolated value divided by the square root of 2. Luminex measurements above the upper limit of quantification were set to the upper limit of quantification. Four additional biomarkers (C-reactive protein, prothrombin time, activated partial thromboplastin time, and platelet counts) were measured by the institutional routine laboratory. Details are provided in the Appendix, Table S2.

To characterise the variation in host responses across different infectious aetiologies, we measured 27 biomarkers within four key pathophysiological domains: endothelial and coagulation activation, inflammation and organ damage, cytokine responses, and chemokine responses.^6^^,^^7^ Since our objective was to assess pathogen-specific host responses, we focused our analyses on patients with a known causative agent; plasma biomarker concentrations in sepsis patients with an unknown causative agent are provided in Supplementary Data.

### Statistical analysis

All biomarker data were transformed to a normal distribution using the Box–Cox method prior to statistical analysis (Appendix, Figure S1). Missing biomarker values (Appendix, Table S2) were considered missing at random; these were imputed using multiple imputation by chained equations, applying the classification and regressions tree method with 10 iterations and 10 imputations.^8^ One imputed dataset was randomly selected for further analysis. Biomarker data were scaled prior to Principal Component Analysis (PCA). A correlation matrix of the scaled data is provided in the Appendix (Figure S2). We expressed differences in biomarker concentrations as the Cohen's *d* or Hedges' *g*, a method for describing effect sizes^9^ with 95% confidence intervals based on 2·5th and 97·5th percentile of 2000 bootstrap replicates and calculated statistical significance using Wilcoxon rank sum testing and corrected for multiple testing using the Benjamini-Hochberg (BH) procedure. Differences on the PCA plots between groups were tested by analysis of variance on the first and second principal component. Single groups were then tested against the grand mean of all other categories using Wilcoxon rank sum testing. For the effect size heatmaps, we calculated Cohen's *d* or Hedges' *g* between patient groups and non-infectious ICU patients depending on group size. Cohen's *d* was used for subgroups with more than 25 patients, whereas Hedges' *g* was applied when subgroup sizes were below 25 to account for small-sample bias. Interpretation of effect sizes followed Cohen's original conventions. We applied variance partitioning analysis to quantify the proportion of variance in biomarker responses associated with causative agents, host demographics (age, sex, comorbidities), infection source and disease severity.^10^ All covariates were selected based on prior evidence of their influence on the host immune response and were included as explanatory variables in the analysis. All statistical analyses were performed using R version 4·4·2 (Vienna, Austria).

### Ethics statement

The study was approved by the institutional ethics committee (Kasturba Medical College and Kasturba Hospital Institutional Ethics Committee: IEC: 371/2018; approval date: 12/06/2018), Institutional Biosafety Committee, and Health Ministry Screening Committee, Ministry of Health and Family Welfare, Government of India (project approval number 2020–9817). Written consent was obtained from all participants or their legal representative.

### Role of the funding source

Funders did not have any role in study design, data collection, data analyses, interpretation, or writing of the report.

## Results

**Fig. 1:**
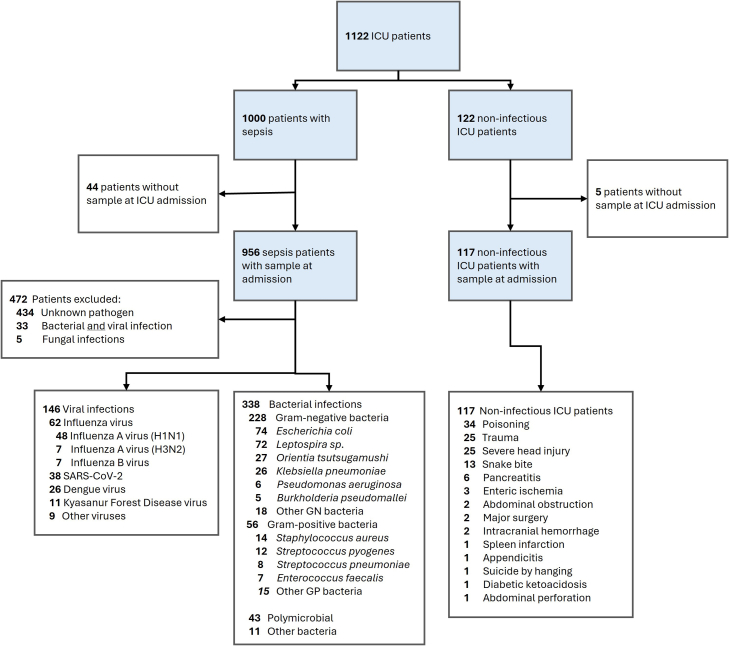
**Study flowchart**.

We enrolled 956 ICU patients hospitalised with sepsis (Table 1) and 117 ICU patients without infection (Fig. 1, Table S3). Bacteria were the causative agent in 338 patients (35·4%), virus in 146 patients (15·3%), 33 patients had a bacterial plus viral infection (3·5%), 5 patients were diagnosed with a fungal infection (0·5%) and in 434 patients the microbiological aetiology remained unknown (45·4%). Baseline characteristics and outcomes of sepsis patients with known and unknown aetiology are provided in Table 1 and Table S4 respectively. Disease severity and hospital mortality were comparable between patients with and without a known causative agent (Table S4).

**Table 1:** Baseline characteristics and outcomes of patients with sepsis.

	n = 956
Baseline demographics	
Age, years	55 [44, 65]
Male	637 (66·6)
Female	319 (33·4)
Comorbidities, n (%)	
Any comorbidity	568 (59·4)
Charlson comorbidity index	2·00 [0·00, 3·00]
Cardiovascular disease	129 (13·5)
Hypertension (medicated)	347 (36·3)
Type 2 diabetes mellitus	339 (35·5)
Chronic lung disease	89 (9·3)
Chronic kidney disease	62 (6·5)
Chronic liver disease	64 (6·7)
Malignancy	22 (2·3)
Auto-immune disease	9 (0·9)
Severity	
SOFA score	6·00 [4·00, 9·00]
APACHE 2 score	13·00 [9·00, 19·00]
Septic shock	376 (39·3)
Laboratory values	
White blood cell count, 10ˆ3/μL	11·45 [7·20, 17·20]
Neutrophils, 10ˆ3/μL	8·87 [4·75, 13·81]
Lymphocytes, 10ˆ3/μL	0·86 [0·49, 1·47]
Neutrophil/Lymphocyte ratio	9·51 [5·17, 16·97]
Lactate, mg/dL	23·15 [14·57, 42·42]
Bilirubin, mg/dL	1·16 [0·52, 2·75]
Creatinin, mg/dL	1·75 [1·06, 3·44]
Outcomes	
Received mechanical ventilation	439 (46·1)
Length of mechanical ventilation, days	2·30 [1·17, 5·02]
Received renal replacement therapy	166 (21·8)
In-hospital mortality	232 (24·3)
3 month mortality	278 (29·1)
Length of ICU stay, days	4·53 [2·54, 8·24]
Length of hospital stay, days	8·67 [4·70, 13·72]

Continuous data are presented a*s* median [interquartile range], and categorical data are presented as number (percentages). Abbreviations: APACHE, Acute Physiology And Chronic Health Evaluation; ICU, Intensive care unit; SOFA, Sequential Organ Failure Assessment.

**Fig. 2:**
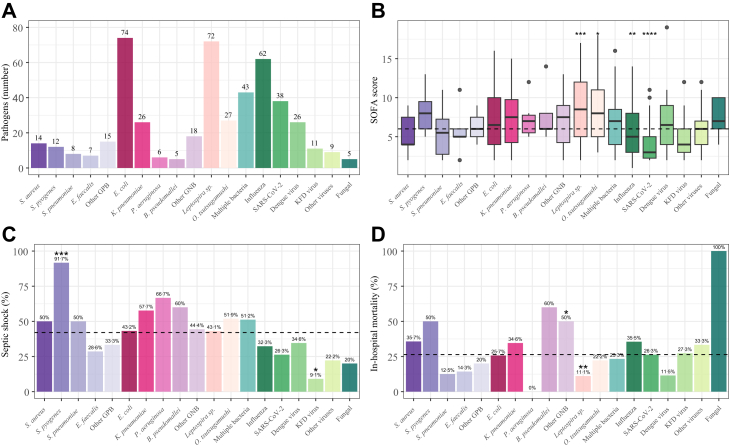
**Distribution of causative pathogens (A), and SOFA scores (B), septic shock (C) and mortality (D) across pathogen groups**. **(A)** Bar chart depicting the number of patients **(B)** Boxplot depicting SOFA scores per pathogen type **(C and D)** Bar chart depicting the percentage of patients with septic shock and in hospital mortality across pathogens. The mean value for all included sepsis patients is shown as a horizontal dotted line. Statistical testing was performed by comparing each pathogen group against this overall mean. ∗P < 0·05, ∗∗P < 0·01, ∗∗∗P < 0·001, ∗∗∗∗P < 0·0001. Abbreviations: GPB, Gram-positive bacteria; GNB, “classical” Gram-negative bacteria; KFD virus, Kyasanur Forest Disease virus; SOFA, Sequential Organ Failure Assessment.

The most prevalent microorganism was *Escherichia (E.) coli* (n = 74), followed by *Leptospira* species (n = 72) and influenza (n = 62); other microorganisms in this monomicrobial group are summarised in Fig. 2A. Fig. 2B, C and D shows pathogen distribution with disease severity (SOFA score, shock) and in-hospital mortality. Hospital mortality was highest in those infected with *S. pyogenes* (50%), followed by *S. aureus* (35·7%) and influenza (35·5%); sepsis caused by *Leptospira* sp. or dengue virus was associated with a relatively low hospital mortality (11·1% and 11·5% respectively).

**Fig. 3:**
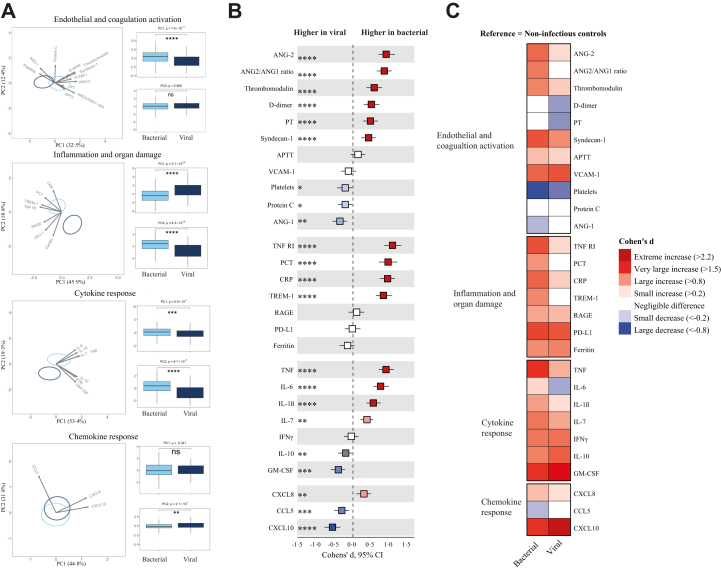
**Diverging host response signatures in response to bacterial and viral pathogens**. **(A)** Principal component analysis (PCA) of biomarkers per pathophysiological domain. X-axis and Y-axis shows the percentage of variance explained by PC1 and PC2 respectively. The ellipses illustrate the most central 10% of patients for each group, centred on the group mean position. The arrows indicate the direction (arrow orientation) and strength (arrow length) of the correlation between each biomarker and the PCs. Box plots display PC1 and PC2 distributions. Light blue indicates bacterial and dark blue indicates viral sepsis **(B)** The X-axis depicts effect sizes (Cohen's *d*). The colour of the squares corresponds to the biomarkers that are significantly higher (red) or significantly lower (blue) in bacterial sepsis vs viral sepsis. A darker shade of the squares corresponds to a higher significance level **(C)** Heatmap depicting the Cohen's *d* between bacterial and viral pathogen groups with non-infectious ICU controls as reference. ∗P < 0·05, ∗∗P < 0·01, ∗∗∗P < 0·001, ∗∗∗∗P < 0·0001. Abbreviations: ANG-1, angiopoietin-1; ANG-2, angiopoietin-2; VCAM-1, vascular cell adhesion molecule-1; PT, prothrombin time; APTT, activated partial thromboplastin time; RAGE, receptor for advanced glycation end-products; TNFR1, tumour necrosis factor receptor I; TREM-1, triggering receptor expressed on myeloid cells-1; PD-L1, programmed death-ligand 1; PCT, procalcitonin; CRP, C-reactive protein; IL, interleukin; TNF, tumour necrosis factor; GM-CSF, granulocyte-macrophage colony-stimulating factor; IFN-γ, interferon-γ; CCL, C–C motif chemokine ligand; CXCL, C-X-C motif chemokine ligand.

PCA per domain revealed significant group separation across all domains (Fig. 3A). At individual marker levels (unadjusted analysis), 22 biomarkers were significantly different between patients with bacterial and viral sepsis, across all pathophysiological domains (BH-adjusted P < 0·05; 7 higher in viral sepsis, 15 higher in bacterial sepsis; Fig. 3B). Bacterial sepsis was associated with stronger coagulation activation (higher D-dimer levels, lower platelet counts and lower protein C concentrations, and prolonged prothrombin time), disturbed endothelial barrier function (higher angiopoietin-2 and lower angiopoietin-1), endothelial glycocalyx degradation (higher syndecan-1), and elevated levels of multiple inflammatory, cytokine and chemokine markers. Notable exceptions were granulocyte-macrophage colony-stimulating factor (GM-CSF) and the chemokines CCL5 and CXCL10. When using non-infectious ICU controls as reference, patients with either bacterial or viral sepsis had biomarker profiles consistent with more disturbances across all domains (Fig. 3C). Plasma biomarker concentrations in sepsis patients with and without an unknown causative agent are provided in Table S5.

**Fig. 4:**
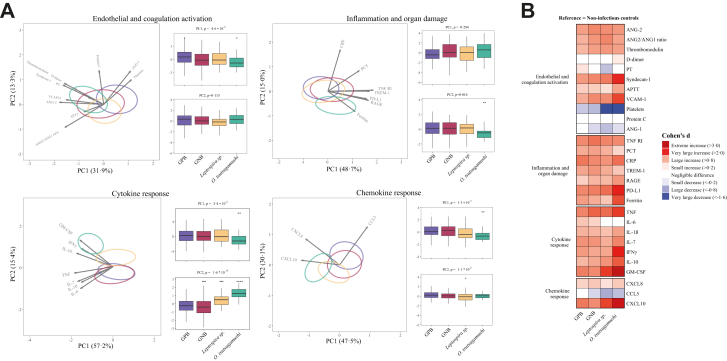
**Different bacterial causes of sepsis elicit distinct host response profiles across all pathophysiological domains**. **(A)** Principal component analysis (PCA) of biomarkers per pathophysiological domain. X-axis and Y-axis shows the percentage of variance explained by PC1 and PC2 respectively. The ellipses illustrate the central 10% of patients for each group, centred on the group mean position. The arrows indicate the direction (arrow orientation) and strength (arrow length) of the correlation between each biomarker and the PCs. Box plots display PC1 andPC2 distributions. **(B)** Heatmap depicting the Cohen's *d* between pathogen groups with non-infectious ICU controls as reference. GPB, Gram-positive bacteria, GNB, “classical” Gram-negative bacteria. For biomarker abbreviations see legend of Fig. 2.

Sepsis caused by *O. tsutsugamushi* was associated with the strongest host response changes across multiple domains (Fig. 4A) relative to gram-positive bacteria, “classical” gram-negative bacteria, and the atypical gram-negative pathogens *Leptospira* sp. particularly reflected in elevated levels of vascular cell adhesion molecule-1, syndecan-1 and high ferritin and soluble programmed death-ligand (PD-L1) levels (Fig. 4B). *O. tsutsugamushi* sepsis was also associated with enhanced proinflammatory and anti-inflammatory cytokine responses. Plasma IL-6 were lower in sepsis caused by *O*. *tsutsugamushi* or *Leptospira* sp. relative to sepsis caused by “classical” Gram-positive or Gram-negative bacteria. D-dimer and protein C levels (reflecting coagulation activation) were not different from non-infectious ICU control patients, irrespective of bacterial sepsis subgroup.

**Fig. 5:**
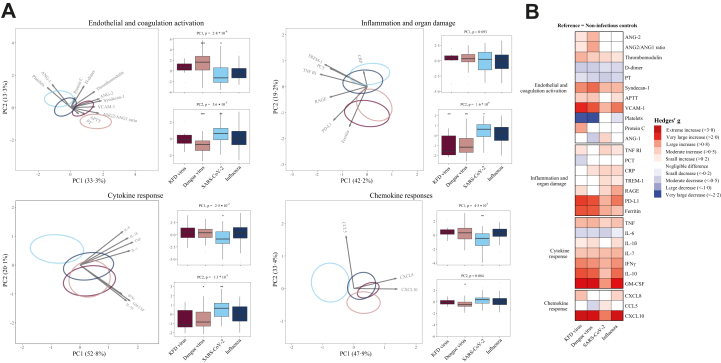
**Sepsis caused by viral haemorrhagic fevers and respiratory viruses is associated with distinct host response profiles**. **(A)** Principal component analysis (PCA) of biomarkers per pathophysiological domain. Principal components (PC) 1 and 2 are plotted per domain. The X-axis label shows the percentage of variance explained by PC1, and the Y-axis label shows the percentage of variance explained by PC2. The ellipse indicates the central 10% of the groups. The arrows indicate the direction (arrow orientation) and strength (arrow length) of the correlation between each biomarker and the PCs. Box plots display PC1 and PC2 distributions **(B)** Heatmap depicting the Hedges' *g* between the disease groups across pathophysiological domains with non-infectious ICU controls as reference. For abbreviations see legend of Fig. 2.

Unadjusted PCA per domain for viral sepsis indicated a clear distinction between viral haemorrhagic fevers and respiratory viruses, particularly for the endothelial and coagulation domain, and the inflammation and organ damage domain (Fig. 5A). These were driven by elevated levels of biomarkers in viral haemorrhagic fevers, indicating greater endothelial barrier function disruption (angiopoietin-2), glycocalyx degradation (syndecan-1) and endothelial activation (VCAM-1) (Fig. 5A). Separation in the inflammation and organ damage domain was mainly driven by ferritin and soluble PD-L1 concentrations. At individual biomarker levels, using non-infectious ICU patients as the reference group, dengue virus was associated with the highest angiopoietin-2 and syndecan-1 concentrations, while ferritin and soluble PD-L1 were equally high in dengue and KFD viral sepsis (Fig. 4B). Soluble receptor for advanced glycation end-products (RAGE) was particularly elevated in influenza. Plasma D-dimer levels were lower in all viral sepsis cases as compared to non-infectious ICU patients. Cytokine and chemokine responses were largely similar between dengue, KFD and influenza, while SARS-CoV-2 stood out for its relatively lesser cytokine/chemokine signature, especially with lower IL-6, IFN-γ, GM-CSF, IL-10 and CXCL10 levels.

**Fig. 6:**
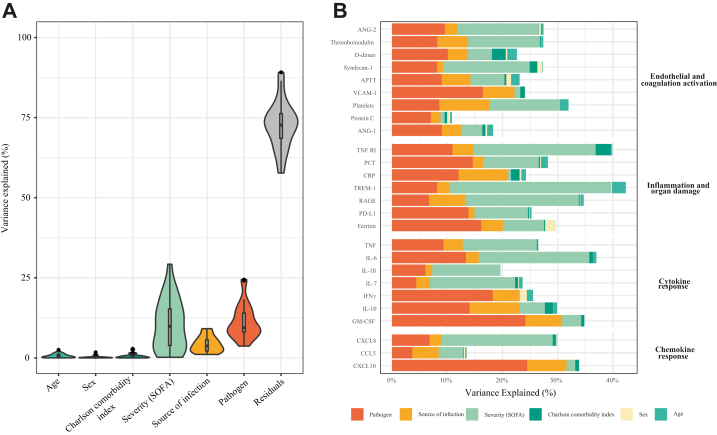
**The causative pathogen explains a large proportion of the variance in host response profiles in patients with sepsis**. **(A)** Violin plots illustrating variance in plasma biomarkers explained by different covariates in sepsis patients with a confirmed causative pathogen. Using a multivariate model for each biomarker (level as outcome), each explanatory covariate was adjusted for the others. The X-axis depicts each covariate that explains the variation of all 27 biomarkers. The Y-axis depicts the total percentage of variance explained for each biomarker per covariate. The box plots inside the violin plots represent the interquartile range and the middle line is the median **(B)** The Y-axis depicts all biomarkers across pathophysiological domains. The X-axis shows the proportion of explained variance of the biomarker indicated by age, sex, comorbidities, severity (SOFA-score), source of infection and pathogen type. Abbreviations: CCI, Charlson comorbidity index; SOFA, sequential organ failure assessment. For biomarker abbreviations see legend of Fig. 2.

The pathogen explained a median of 34·5% of the summed explained biomarker variance excluding residual variance, or a median of 9·4% of the total variance; other contributing factors were the SOFA score (36·1% and 9·9% respectively) and the source of infection (13·6% and 3·7%) (Fig. 6A). Individual biomarkers for which the pathogen explained a particularly large proportion of the variance included CXCL10 (24·4%), GM-CSF (24·1%), IFN-γ (18·2%), VCAM-1 (16·5%), ferritin (16·1%), procalcitonin (14·6%), IL-10 (14·0%), and soluble PD-L1 (13·8%) when corrected for other covariates (Fig. 6B).

We performed several sensitivity analyses (Tables S6–S9). In addition, we repeated all primary and sensitivity analyses with adjustment for age, sex, and disease severity. Although statistical significance changed for a limited number of comparisons, the overall direction and main expression patterns remained consistent with the primary analysis.

## Discussion

In this study, we sought to characterise the host immune response across diverse aetiologies of sepsis in a tertiary care centre in India. While patients were enrolled based on the current Sepsis-3 criteria,^1^ our cohort differed from sepsis populations from high-income countries, with causative organisms comprising both pathogens common to HICs and LMICs. We observed a marked heterogeneity in host response profiles not only between bacterial and viral sepsis, but also within these categories. *O*. *tsutsugamushi* elicited a response distinct from other bacterial pathogens, while within the viral group, there was a clear divergence between haemorrhagic fever viruses and respiratory viruses. Our analyses indicate that the causative pathogen is a key determinant of the host response in sepsis patients in whom the infectious agent could be identified. These observations highlight the complexity of host–pathogen interactions in sepsis.

Unlike most previous studies, which focused on a limited set of biomarkers or examined one pathogen group at a time,^11^ we simultaneously investigated several pathophysiological domains and included a broad spectrum of bacterial and viral pathogens. Additionally, we report this wide biomarker panel in patients with KFD, an infection not studied previously. This approach allowed us to uncover distinct pathogen-specific host response patterns, accentuating underlying differences that would remain obscured in more narrowly focused analyses.

Bacterial sepsis, relative to viral sepsis, was associated with an overall more pronounced host response entailing endothelial and coagulation activation, systemic inflammation and cytokine release. Importantly, however, our study demonstrates that within these broad etiologic categories, host responses vary considerably between distinct pathogens. Among bacterial infections, *O. tsutsugamushi*, the causative agent of scrub typhus, induced the most profound alterations. This microorganism is an obligate intracellular bacterium that can strongly activate inflammasomes and release of pro-inflammatory cytokines, driving robust systemic inflammation.^11^ The host response to *Leptospira* differed from that associated with sepsis caused by “classical” bacterial pathogens, characterised by more severe thrombocytopaenia, and markedly higher levels of CXCL10 and GM-CSF. While these host response features have been reported previously in leptospirosis,^12^^,^^13^ thus far, comparison of host responses in a cohort of concurrently enrolled sepsis patients with other aetiologies was lacking. One possible explanation is that *Leptospira* activates the immune system through a different pathway. Unlike most Gram-negative bacteria, its lipopolysaccharide is not recognised by Toll-like receptor (TLR) 4; instead, *Leptospira* is sensed primarily via TLR2, which detects outer membrane lipoproteins such as LipL32.^14^ TLR2 activation has been implicated in the induction of type I interferons, which may help explain the elevated CXCL10 levels.^14^

Among viral sepsis, dengue and KFD caused by distinct haemorrhagic viruses of the Flaviviridae family were associated with stronger host response changes compared with those observed in respiratory sepsis caused by SARS-CoV-2 or influenza. Dengue virus can activate immune cells through multiple pattern recognition receptors, including TLRs, C-type lectin receptors and RIG-I-like receptors, resulting in cytokine release and direct endothelial cell damage.^15^ Immune evasion strategies of dengue virus allow high viral loads that amplify inflammatory and coagulation pathways, exacerbating tissue damage. KFD is a tick-borne illness of which the host immune response remains largely uncharacterised.^16^ The current investigation indicates that KFD and dengue induce similar host response alterations across multiple pathophysiological domains. Viral components shared by dengue and KFD virus, including their positive-sense single-stranded RNA genomes, the capsid, membrane, and envelope proteins, as well as several conserved non-structural proteins (NS1 through NS5), may partly explain overlapping systemic immune responses.^17^

With regard to individual biomarkers, our finding of higher C-reactive protein, procalcitonin and IL-6, and lower CXCL10 concentrations in bacterial relative to viral sepsis is in agreement with earlier investigations.^18^ While elevated CXCL10 levels, an interferon-stimulated chemokine, in viral sepsis aligns with its critical role in recruiting immune cells during antiviral responses,^19^ it should be noted that the highest CXCL10 levels were observed in *O. tsutsugamushi* and *B. pseudomallei* infections. These intracellular bacteria may activate cytosolic pattern recognition receptors within host cells, unlike most extracellular bacterial infections, which are sensed via surface receptors.^11^^,^^20^ Strong upregulation of CXCL10 has been shown in other intracellular non-viral diseases, such as tuberculosis, malaria and *Clostridium difficile* infections.^21^, ^22^, ^23^ Previous studies reported that combining C-reactive protein, procalcitonin, and CXCL10 improves the distinction between viral and bacterial infections compared to using either biomarker individually.^24^^,^^25^ The present study, while not focused on diagnostic biomarkers, may have important implications for future diagnostic strategies that include CXCL10, particularly in LMICs, where intracellular bacterial infections are more prevalent.

We used variance partitioning analysis to quantify the proportion of variance in host response biomarkers explained by pathogen type, while adjusting for baseline characteristics such as age, sex, comorbidities, SOFA score, and infection source, and confirmed that pathogen identity is a major contributor. Importantly, the magnitude of this pathogen-driven variation was comparable to that explained by disease severity, highlighting the combined influence of microbial factors and the host clinical status on the immune response. These findings accentuate the potential of integrating pathogen-specific biomarker signatures with severity indicators to inform biomarker-guided therapeutic strategies tailored to individual patients. Nonetheless, we acknowledge the possibility that unmeasured or residual confounding factors may influence biomarker variability. Our analyses were limited to sepsis patients in whom a causative pathogen was detected, which may limit extrapolation to culture-negative cases. Therefore, further validation in additional cohorts is warranted.

Our study has limitations. By focussing on patients with mono-microbial infections, we were able to isolate the host response associated with specific pathogens. We did not account for variation in pathogen virulence factors, which could help explain additional variance in host responses and further refine pathogen-specific biomarker signatures. Additionally, undetected co-infections could have influenced the observed patterns. While such misclassification might seem problematic, it would likely reduce the apparent differences between groups rather than exaggerate them, making our reported pathogen-specific differences conservative. Several pathogen-specific subgroups were relatively small, which increases statistical uncertainty and limits the precision of effect estimates. These findings should therefore be interpreted with caution and should be confirmed in larger cohorts. Additionally, pathogen assignment occurred within routine clinical care, where initial laboratory findings may have influenced the diagnosis. Another limitation was the low number of patients with community-acquired fungal infections, which precluded meaningful fungal-specific analyses. In 45·4% of patients a causative pathogen could not be identified; it is uncertain whether “missed” pathogens in this group impacted analyses of pathogen-specific biomarker profiles. Our study did not seek to develop a diagnostic biomarker-based test; such tests would be difficult to implement in clinical practice in LMICs due to financial constraints.

In summary, our investigation offers a nuanced assessment of the systemic host response among different aetiologies of sepsis, providing insight into alterations in distinct pathophysiological pathways within a clinically relevant, yet relatively unexplored geographic region. These findings demonstrate that sepsis heterogeneity extends beyond the conventional bacterial-viral dichotomy, with distinct pathogens eliciting divergent responses across multiple host response domains. Although our data do not provide an immediately applicable diagnostic tool to discriminate aetiology at the bedside, they offer a biological framework for understanding pathogen-specific dysregulation. Such insights may guide future biomarker selection and the development of diagnostic strategies aimed at refining aetiological classification. Our results suggest that future interventional trials may benefit from incorporating pathogen-specific designs that reflect underlying biological diversity.

## Contributors

H.S.V., C.M., W.J.W., and T.v.d.P were involved in study conception, design, and supervision. V.A.E., H.S.V., and C.M. were involved in obtaining ethical and legal authorisations. J.J.B., V.A.E., H.S.V., A.A., A.G., and A.M.K acquired the data. H.P.S. and J.M.B provided statistical guidance and guided biomarker measurements. J.J.B. and V.A.E. had full access to the data and take full responsibility for the integrity of the data and the accuracy of the data analysis. J.J.B. and T.v.d.P. were involved in the data interpretation. J.J.B. and T.v.d.P. draughted the manuscript, and all authors revised it critically for the important intellectual content. All authors gave final approval of this version.

## Data sharing statement

The data presented in this manuscript can be made available upon reasonable request to the corresponding author.

## Declaration of interests

Dr. Peters-Sengers was supported by the Dutch Kidney Foundation (Nierstichting) postdoc KOLFF grant 19OK009. Dr. Wiersinga reports grants from the Netherlands Organisation for Health Research and Development (ZonMw), EU/Eurostars and Moderna outside the submitted work. Dr. van der Poll reports grants from Immunexpress, EU/Horizon 2020 (FAIR, Immunosep), the Ministry of Economic Affairs & Health Holland, and the Dutch Thrombosis Foundation, as well as a consultancy with Matisse (all paid to the institution); he is a member of Data Safety Monitoring Board of REMAP-CAP (no payment). Biemond, Earny, Virk, Adukkadukkam, Girish, Klarenbeek, Dr. Butler, and Dr. Mukhopadhyay have no competing interests to declare.
