# Supplementary material for: Host response signatures across sepsis aetiologies in India: a single centre observational study
Source: Lancet Reg Health Southeast Asia. 2026 Mar 21;47:100752. doi: 10.1016/j.lansea.2026.100752 (PMC13022632; doi:10.1016/j.lansea.2026.100752)
Supplement: Supplementary Appendix [file mmc1.docx]

**Distinct Host Response Signatures Across Sepsis Aetiologies in India: a Single-Centre Hospital Based Observational Study**

**SUPPLEMENTARY APPENDIX**

Jason J. Biemond, MD¹, Venkat A. Earny, MSc²ˑ³, Harjeet Virk, MD^4^, Anusree Adukkadukkam, MSc²ˑ³, Anjali Girish, MSc^3·5^, Augustijn M. Klarenbeek¹, Hessel Peters-Sengers, PhD¹ˑ^6^, Joe M. Butler, PhD¹, Chiranjay Mukhopadhyay, PhD²ˑ³, W. Joost Wiersinga, PhD¹ˑ⁷ˑ^8^, Tom van der Poll, PhD¹ˑ⁷ˑ^8^

¹Centre for Infection and Molecular Medicine, Amsterdam University Medical Centre, Location AMC, University of Amsterdam, Amsterdam, the Netherlands; ²Department of Microbiology, Kasturba Medical College, Manipal Academy of Higher Education, Manipal, India; ³Centre for Emerging and Tropical Diseases (CETD), Manipal Academy of Higher Education, Manipal, India; ^4^Department of Microbiology, Queen Alexandra Hospital, Portsmouth Hospitals University National Health Service Trust, Portsmouth, United Kingdom; ^5^Manipal Institute of Virology, Manipal Academy of Higher Education, Manipal, India; ^6^Department of Epidemiology and Data Science, Amsterdam University Medical Centre, Location Vrije Universiteit Amsterdam, Amsterdam, the Netherlands; ^7^Amsterdam Institute for Immunology and Infectious Diseases, Amsterdam University Medical Centre, Amsterdam, the Netherlands; ^8^Division of Infectious Diseases, Department of Medicine, Amsterdam University Medical Centre, University of Amsterdam, Amsterdam, the Netherlands

**Table of contents**

[**Supplementary tables** 3](#_Toc222329138)

[Table S1: Overview of microbiological test characteristics 3](#_Toc222329139)

[Table S2. Biomarkers: stratification according to pathophysiological domain and missing data 4](#_Toc222329140)

[Table S3. Baseline characteristics and outcomes of non-infectious ICU patients 5](#_Toc222329141)

[Table S4. Baseline characteristics and outcomes across patients with bacterial, viral, mixed, fungal or an unknown causative agent 6](#_Toc222329142)

[Table S5. Plasma biomarker concentrations in patients with known and unknown causative pathogens 7](#_Toc222329143)

[Table S6. Comparison of biomarker concentrations between bacterial and viral aetiology: main and four sensitivity analyses 8](#_Toc222329144)

[Table S7. Comparison of biomarker concentrations between bacterial aetiologies unadjusted and adjusted for age, sex, and severity: main and four sensitivity analyses 9](#_Toc222329145)

[Table S8. Comparison of biomarker concentrations between viral aetiologies unadjusted and adjusted for age, sex, and severity: main and four sensitivity analyses 10](#_Toc222329146)

[Table S9. Comparison of biomarker concentrations between bacterial and viral aetiology corrected for age, sex, and severity: main and four sensitivity analyses 11](#_Toc222329147)

[**Supplementary figures 12**](#_Toc222329148)

[Supplemental Figure 1. Biomarker distribution plots after Box-Cox transformation 12](#_Toc222329149)

[Supplementary Figure 2. Correlation matrix of the scaled data underlying the PCA analyses. 13](#_Toc222329151)

[Figure S3. Comparison of host response profiles induced by O. tsutsugamushi with those induced by the two most common “classical” pathogens (E. coli and K. pneumoniae). 14](#_Toc222329152)

# **Supplementary tables**

# Table S1: Overview of microbiological test characteristics

| **Test** | **Kit** |
| --- | --- |
| **Microscopy** |  |
| *Cryptococcus* | India ink (KOH-1-NOOR 3080 F Universal or Becton Dickinson M7-74811) |
| *Vibrio cholerae* | Hanging drop: Normal saline, Pure isolated colony |
| **Bacterial diagnostics** |  |
| Bacteria | Conventional cultures (Aerobic and anaerobic) |
| *Burkholderia pseudomallei* (PCR) | In-house PCR: |
|  | 1) QIAamp DNA Mini Kit |
|  | 2) HotStarTaq Master Mix Kit |
|  | 3) Taqman Primers |
|  | 4) TrackIt™ 100 bp DNA Ladder |
| *Leptospira sp.* (IgM ELISA) | Jmitra; Lepto IgM Microlisa |
| *Orientia tsutsugamushi* (IgM ELISA) | INBIOS; Scrub Typhus Detect IgM ELISA |
| Orientia tsutsugamushi (IgM Micro Immunofluorescence) | Fuller Laboratories; Orientia MIF IgM Kit |
| **Human herpes viruses** |  |
| Herpes simplex virus | RealStar® HSV PCR Kit 1.0 |
| Varicella zoster virus | RealStar® VZV PCR Kit 1.0 |
| Cytomegalovirus | RealStar® CMV PCR Kit 1.0 |
| Epstein-barr virus | RealStar® EBV PCR Kit 2.0 |
| Respiratory viruses |  |
| Human metapneumovirus (PCR) | In-house PCR (Fusion gene) |
| SARS-CoV-2 | Meril COVID-19 One-step RT-PCR Kit |
| Influenza virus | RealStar® Influenza Screen & Type RT-PCR Kit 4.0 |
|  | In-house kit |
| **Viral diagnostics** |  |
| Kyasanur forest disease virus | In-house PCR (NS-5 region) |
| Japanese Encephalitis virus | NIV - Japanese Encephalitis IgM capture ELISA |
| Dengue virus | Panbio Dengue IgM capture ELISA |
|  | Panbio Dengue Early ELISA |
|  | RealStar® Dengue RT-PCR Kit 2.0 |
| Chikungunya virus (IgM ELISA) | CHIKjj Detect™ IgM ELISA Kit |
| Chikungunya virus (PCR) | RealStar® Chikungunya RT-PCR Kit 2.0 |
| HIV-1 (RNA Quantitative Real-Time PCR) | Xpert HIV-1 viral load |
| HIV (Antibodies-Chemiluminescence) | VITROS; HIV Combo Reagent Pack |
| **Fungal diagnostics** |  |
| Fungi | Conventional culture |
| **Parasital diagnostics** |  |
| Malaria | Quantative buffy coat (QBC) |
| Filariasis | Microfilaria test (Blood smear) |

# Table S2. Biomarkers: stratification according to pathophysiological domain and missing data

| **Biomarker** | **% unmeasured** | **% below LLOQ** | **% above ULOQ** | **% within detection range** |
| --- | --- | --- | --- | --- |
| **Endothelial and coagulation activation** | | | | |
| ANG-1 | 0 | 0·1 | 0 | 99·9 |
| ANG-2 | 0 | 0 | 0·7 | 99·3 |
| Thrombomodulin | 0 | 0 | 0 | 100 |
| VCAM-1 | 0 | 0 | 4 | 96 |
| Syndecan-1 | 0 | 0 | 0·2 | 99·8 |
| D-dimer | 0 | 0 | 0·4 | 99·6 |
| Protein C | 0 | 0·1 | 0 | 99·9 |
| Platelets | 3·9 | - | - | - |
| PT | 28·0 | - | - | - |
| APTT | 30·1 | - | - | - |
| **Inflammation and organ damage** | | | | |
| RAGE | 0 | 0 | 0·2 | 99·8 |
| Ferritin | 0 | 0 | 2·3 | 97·7 |
| TNFR1 | 0 | 0 | 0 | 100 |
| TREM-1 | 0 | 0·1 | 0 | 99·9 |
| PD-L1 | 0 | 0 | 0 | 100 |
| PCT | 0 | 0 | 22·6 | 77·4 |
| CRP | 40·0 | - | - | - |
| **Cytokine response** | | | | |
| IL-1β | 0 | 2 | 0 | 98 |
| IL-6 | 0 | 0·1 | 0·6 | 99·4 |
| IL-7 | 0 | 0·1 | 0 | 99·9 |
| TNF | 0 | 0 | 0 | 100 |
| GM-CSF | 0 | 0 | 0 | 100 |
| IFN-γ | 0 | 0·5 | 0 | 99·5 |
| IL-10 | 0 | 8·2 | 0 | 91·8 |
| **Chemokine response** | | | | |
| CCL5 | 8·7 | 0·2 | 0 | 91 |
| CXCL8 | 0 | 0 | 1·8 | 98·2 |
| CXCL10 | 0 | 0 | 14·3 | 85·7 |

Percentage of samples with unmeasured values· below the lower limit of quantification (LLOQ)·or above the upper limit of quantification (ULOQ) ) for each biomarker. The last column indicates the percentage of samples that were measured and quantifiable (% within the detection range). Biomarkers are grouped by pathophysiological domain. Abbreviations: ANG-1: angiopoietin-1; ANG-2: angiopoietin-2; VCAM-1: vascular cell adhesion molecule-1; PT: prothrombin time; APTT: activated partial thromboplastin time; RAGE: receptor for advanced glycation end-products; TNFR1: tumour necrosis factor receptor I; TREM-1: triggering receptor expressed on myeloid cells-1; PD-L1: programmed death-ligand 1; PCT: procalcitonin; CRP: C-reactive protein; IL: interleukin; TNF: tumour necrosis factor; GM-CSF: granulocyte-macrophage colony-stimulating factor; IFN-γ: interferon-γ; CCL: C-C motif chemokine ligand; CXCL: C-X-C motif chemokine ligand.

# Table S3. Baseline characteristics and outcomes of non-infectious ICU patients

|  | **Non-infectious ICU cohort** |
| --- | --- |
| **Baseline demographics** | **n = 117** |
| Age, years | 41 [28, 60] |
| Sex (male) | 94 (80·3) |
| **Comorbidities, n (%)** |  |
| Any comorbidity | 28 (23·9) |
| Charlson comorbidity index | 0 [0, 2] |
| Cardiovascular disease | 4 (3·4) |
| Hypertension (medicated) | 15 (12·8) |
| Type 2 Diabetes mellitus | 15 (12·8) |
| Chronic lung disease | 3 (2·6) |
| Chronic kidney disease | 1 (0·9) |
| Chronic liver disease | 1 (0·9) |
| Malignancy | 0 (0·0) |
| Auto-immune disease | 0 (0·0) |
| **Severity** |  |
| SOFA score | 4 [2, 6] |
| APACHE 2 score | 12 [6, 16] |
| Shock | 35 (29·9) |
| **Laboratory values** |  |
| White blood cell count, 10^3/μL | 11·30 [8·95, 15·35] |
| Neutrophils, 10^3/μL | 8·88 [6·57, 13·68] |
| Lymphocytes, 10^3/μL | 1·08 [0·70, 1·69] |
| Neutrophil/Lymphocyte ratio | 8·90 [4·79, 15·79] |
| Lactate, mg/dL | 23·50 [15·70, 40·60] |
| Bilirubin, mg/dL | 0·68 [0·46, 1·37] |
| Creatinin, mg/dL | 1·01 [0·82, 1·41] |
| **Outcomes** |  |
| Received ventilation | 70 (59·8) |
| Length of mechanical ventilation | 1·98 [0·79, 4·93] |
| Inotropic support | 45 (38·5) |
| Received renal replacement therapy | 5 (6·8) |
| In-hospital mortality | 19 (16·2) |
| 3 month mortality | 22 (18·8) |
| Length of ICU stay | 4·84 [2, 8·14] |
| Length of hospital stay | 9·10 [4·59, 14·65] |

Continuous data are presented a*s* median [interquartile range] and categorical data are presented as number (percentages). Abbreviations: APACHE: Acute Physiology And Chronic Health Evaluation; ICU: Intensive care unit; SOFA: Sequential Organ Failure Assessment

# Table S4. Baseline characteristics and outcomes across patients with bacterial, viral, mixed, fungal or an unknown causative agent

|  | **Stratified by pathogen** | | | | |
| --- | --- | --- | --- | --- | --- |
|  | **Bacterial** | **Viral** | **Mixed** | **Fungal** | **Unknown pathogen** |
| **Baseline demographics** | **n = 338** | **n = 146** | **n = 33** | **n = 5** | **n = 434** |
| Age, years | 54·50 [44, 63] | 53·50 [41·25, 66] | 49·00 [34, 60] | 64 [50, 69] | 57·50 [45, 67] |
| Sex (male) | 232 (68·6) | 94 (64·4) | 22 (66·7) | 5 (100·0) | 284 (65·4) |
| **Comorbidities** |  |  |  |  |  |
| Any comorbidities | 187 (55·3) | 79 (54·1) | 15 (45·5) | 4 (80·0) | 283 (65·2) |
| Charlson comorbidity index | 2 [0, 3] | 2 [0, 3] | 1·00 [0, 3] | 3 [1, 6] | 2 [1, 3] |
| Cardiovascular disease | 34 (10·1) | 19 (13·0) | 5 (15·2) | 1 (20·0) | 70 (16·1) |
| Hypertension (medicated) | 102 (30·2) | 52 (35·6) | 10 (30·3) | 1 (20·0) | 182 (41·9) |
| Type 2 Diabetes mellitus | 122 (36·1) | 48 (32·9) | 9 (27·3) | 2 (40·0) | 158 (36·4) |
| Chronic lung disease | 15 (4·4) | 14 (9·6) | 4 (12·1) | 0 (0·0) | 56 (12·9) |
| Chronic kidney disease | 15 (4·4) | 10 (6·8) | 0 (0·0) | 2 (40·0) | 35 (8·1) |
| Chronic liver disease | 31 (9·2) | 3 (2·1) | 2 (6·1) | 0 (0·0) | 28 (6·5) |
| Malignancy | 6 (1·8) | 4 (2·7) | 2 (6·1) | 1 (20·0) | 9 (2·1) |
| Auto-immune disease | 4 (1·2) | 0 (0·0) | 1 (3·0) | 0 (0·0) | 4 (0·9) |
| **Severity** |  |  |  |  |  |
| SOFA score | 7 [4, 10] | 4·50 [3, 8] | 7 [5, 9] | 7 [6, 10] | 6 [4, 9] |
| APACHE 2 score | 14 [9, 20] | 10 [7, 17·75] | 13 [8, 15] | 16 [4, 17] | 14 [9, 20] |
| Septic shock | 161 (47·6) | 42 (28·8) | 12 (36·4) | 1 (20·0) | 160 (36·9) |
| **Laboratory values** |  |  |  |  |  |
| White blood cell count, 10^3/μL | 12·95 [8, 18·42] | 8·60 [4·40, 13·40] | 8·50 [3·77, 15·45] | 7 [4·40, 16] | 11·70 [8·12, 17·80] |
| Neutrophils, 10^3/μL | 9·70 [5·20, 14·30] | 6·38 [3·17, 11·01] | 6·75 [2·35, 10·90] | 5·68 [3·45, 13·86] | 9·23 [5·54, 14·73] |
| Lymphocytes, 10^3/μL | 0·79 [0·45, 1·55] | 0·76 [0·48, 1·22] | 0·72 [0·35, 1·87] | 0·42 [0·33, 0·78] | 0·92 [0·56, 1·47] |
| Neutrophil/Lymphocyte ratio | 10·25 [5·42, 18·99] | 7·07 [3·78, 13·18] | 5·28 [2·70, 10·21] | 12·37 [8·16, 23·03] | 9·62 [5·50, 17·19] |
| Lactate, mg/dL | 27·30 [16·92, 48·48] | 18·20 [12·05, 26·95] | 20·60 [14·45, 65·95] | 12·70 [10·10, 16·40] | 23·15 [14·57, 41·60] |
| Bilirubin, mg/dL | 1·82 [0·79, 4·60] | 0·58 [0·33, 1·19] | 1·77 [0·70, 4·35] | 0·73 [0·65, 1·44] | 1·01 [0·47, 2·29] |
| Creatinin, mg/dL | 2·24 [1·32, 3·60] | 1·10 [0·80, 1·61] | 1·24 [0·92, 3·23] | 3·79 [1·99, 4·19] | 1·82 [1·09, 3·65] |
| **Outcomes** |  |  |  |  |  |
| Received ventilation | 154 (45·7) | 71 (48·6) | 15 (45·5) | 4 (80·0) | 195 (45·1) |
| Length of mechanical ventilation | 2·36 [1·09, 4·60] | 4·10 [1·86, 8·27] | 2·68 [1·17, 5·55] | 3·07 [2·42, 5·03] | 1·94 [1·05, 3·60] |
| Received renal replacement therapy | 69 (24·3) | 21 (18·8) | 5 (19·2) | 2 (40·0) | 69 (20·7) |
| In-hospital mortality | 82 (24·3) | 41 (28·1) | 9 (27·3) | 5 (100·0) | 95 (21·9) |
| 3 month mortality | 94 (27·8) | 48 (32·9) | 10 (30·3) | 5 (100·0) | 121 (27·9) |
| Length of ICU stay | 4·79 [2·73, 8·90] | 5·52 [3·20, 9·82] | 5·79 [2·33, 7·86] | 4·65 [4·48, 8·54] | 3·94 [2·25, 7·40] |
| Length of hospital stay | 9·74 [5·77, 14·37] | 8·07 [5·15, 12·86] | 9·72 [5·45, 14·39] | 4·65 [4·48, 8·54] | 7·85 [4·21, 13·11] |

# Table S5. Plasma biomarker concentrations in patients with known and unknown causative pathogens

|  | **Causative pathogen known** | **Unknown pathogen** |  |
| --- | --- | --- | --- |
|  | **n = 522** | **n = 434** | **P-value** |
| **Endothelial and coagulation activation** | |  |  |
| ANG-1, pg/mL | 1478.13 [865.09, 3029.90] | 1494.14 [934.35, 3753.94] | 0.160 |
| ANG-2, pg/mL | 14231.23 [6019.69, 29085.98] | 11870.83 [5259.15, 24707.69] | 0.089 |
| ANG2/ANG1 ratio | 9.15 [2.65, 26.57] | 6.67 [2.15, 21.63] | 0.033 |
| Thrombomodulin, pg/mL | 11792.93 [8510.83, 17081.80] | 10758.04 [7434.92, 16615.50] | 0.025 |
| VCAM-1, pg/mL | 3362860.81 [1934467.49, 5770509.80] | 2214109.09 [1131167.93, 4063764.63] | <0.001 |
| Syndecan-1, pg/mL | 16165.89 [9147.64, 26606.32] | 12217.40 [5339.79, 21301.43] | <0.001 |
| D-dimer, pg/mL | 19578623.92 [11293875.86, 40781865.06] | 18895616.01 [10699438.43, 39075569.22] | 0.335 |
| Protein C, pg/mL | 79266.86 [58164.87, 106436.99] | 74115.56 [56335.59, 105680.11] | 0.299 |
| Platelets, 10^3/mL | 116.75 [59.62, 208.37] | 172.50 [92.50, 269.00] | <0.001 |
| PT, sec | 12.10 [11.00, 14.27] | 12.20 [11.00, 14.60] | 0.322 |
| APTT, sec | 32.20 [23.90, 41.37] | 29.55 [23.62, 38.37] | 0.002 |
| **Inflammation and organ damage** | |  |  |
| RAGE, pg/mL | 5189.73 [2727.24, 10796.27] | 4780.10 [2606.13, 10266.92] | 0.319 |
| Ferritin, ng/mL | 641.08 [271.03, 1430.45] | 417.76 [181.94, 1192.31] | <0.001 |
| TNF RI, pg/mL | 1799.93 [1123.18, 2777.95] | 1632.70 [1045.60, 2517.06] | 0.012 |
| TREM 1, pg/mL | 784.99 [447.38, 1326.90] | 744.75 [443.61, 1456.84] | 0.843 |
| PD-L1, pg/mL | 274.04 [146.41, 496.41] | 184.81 [94.96, 384.33] | <0.001 |
| PCT, ng/mL | 3.06 [0.86, 5.52] | 2.34 [0.54, 5.23] | 0.029 |
| CRP, pg/mL | 143.67 [66.12, 260.73] | 96.83 [36.19, 195.22] | <0.001 |
| **Cytokine response** | |  |  |
| IL-1β, pg/mL | 32.08 [18.47, 51.12] | 26.55 [16.35, 45.27] | 0.001 |
| IL-6, pg/mL | 99.11 [36.09, 770.73] | 72.75 [27.89, 372.44] | 0.001 |
| IL-7, pg/mL | 9.14 [6.65, 12.24] | 8.06 [5.61, 11.13] | <0.001 |
| IL-10, pg/mL | 12648.21 [733.61, 761492.86] | 1419.11 [161.50, 75720.81] | <0.001 |
| TNF, pg/mL | 49.85 [23.41, 102.16] | 32.50 [19.70, 70.24] | <0.001 |
| GM-CSF, pg/mL | 326.71 [120.57, 1040.79] | 165.13 [66.98, 619.68] | <0.001 |
| IFNγ, pg/mL | 9.68 [5.09, 22.46] | 7.46 [4.10, 14.56] | <0.001 |
| **Chemokine response** | |  |  |
| CCL5, pg/mL | 5367.26 [3502.22, 9260.56] | 5701.98 [3946.50, 10419.12] | 0.048 |
| CXCL8, pg/mL | 60.53 [27.88, 178.63] | 40.09 [17.85, 95.90] | <0.001 |
| CXCL10, pg/mL | 365.85 [116.89, 959.82] | 169.78 [68.71, 700.61] | <0.001 |

# Table S6. Comparison of biomarker concentrations between bacterial and viral aetiology: main and four sensitivity analyses

|  | **Bacterial** | **Viral** | **Main** | **Sensitivity Analysis 1** | **Sensitivity Analysis 2** | **Sensitivity Analysis 3** | **Sensitivity Analysis 4** |
| --- | --- | --- | --- | --- | --- | --- | --- |
|  | **n = 338** | **n = 146** |  |  |  |  |  |
|  | **n = 310** | **n = 129** |  |  |  |  |  |
|  | **n = 328** | **n = 142** |  |  |  |  |  |
|  | **n = 318** | **n = 140** |  |  |  |  |  |
|  | **n = 285** | **n = 130** |  |  |  |  |  |
| **Endothelial and coagulation activation** | |  |  |  |  |  |  |
| ANG-1, pg/mL | 1374·95 [780·24, 2744·36] | 2006·66 [946·32, 3980·49] | < 0·001 | < 0·001 | 0·001 | 0·002 | 0·003 |
| ANG-2, pg/mL | 18027·16 [8321·07, 36663·26] | 7035·34 [3552·41, 14626·56] | < 0·001 | < 0·001 | < 0·001 | < 0·001 | < 0·001 |
| ANG2/ANG1 ratio | 13·10 [4·01, 35·39] | 2·66 [1·27, 10·46] | < 0·001 | < 0·001 | < 0·001 | < 0·001 | < 0·001 |
| Thrombomodulin, pg/mL | 12987·86 [9390·82, 18114·98] | 9274·89 [7274·30, 12690·46] | < 0·001 | < 0·001 | < 0·001 | < 0·001 | < 0·001 |
| VCAM-1, pg/mL | 3319481·83 [1875111·52, 5272360·36] | 3507572·69 [2103930·84, 6439885·48] | 0·24 | 0·27 | 0·20 | 0·21 | 0·30 |
| Syndecan-1, pg/mL | 17540·49 [10187·43, 27825·03] | 12881·10 [6743·34, 21105·41] | < 0·001 | < 0·001 | < 0·001 | < 0·001 | < 0·001 |
| D-dimer, pg/mL | 24330589·09 [12505834·25, 48095099·19] | 13695016·38 [8730868·77, 24011025·94] | < 0·001 | < 0·001 | < 0·001 | < 0·001 | < 0·001 |
| Protein C, pg/mL | 76945·06 [54470·78, 103691·20] | 82722·22 [62220·27, 115062·38] | 0·053 | 0·039 | 0·069 | 0·083 | 0·032 |
| Platelets, 10^3/mL | 109·25 [56·12, 217·25] | 143·50 [88·00, 225·12] | 0·061 | 0·069 | 0·079 | 0·082 | 0·10 |
| PT, sec | 13·70 [11·62, 17·65] | 12·00 [10·83, 14·00] | < 0·001 | 0·001 | 0·001 | 0·004 | < 0·001 |
| APTT, sec | 33·45 [27·60, 41·25] | 31·40 [27·52, 37·52] | 0·11 | 0·066 | 0·097 | 0·29 | 0·115 |
| **Inflammation and organ damage** | |  |  |  |  |  |  |
| RAGE, pg/mL | 5328·04 [2847·75, 9807·44] | 4550·64 [2357·79, 12192·38] | 0·38 | 0·28 | 0·41 | 0·48 | 0·35 |
| Ferritin, ng/mL | 584·86 [264·69, 1195·13] | 705·19 [271·85, 1907·72] | 0·23 | 0·10 | 0·19 | 0·29 | 0·20 |
| TNF RI, pg/mL | 2173·11 [1497·77, 3103·75] | 1096·29 [830·89, 1579·30] | < 0·001 | < 0·001 | < 0·001 | < 0·001 | < 0·001 |
| TREM 1, pg/mL | 896·79 [584·07, 1587·44] | 456·73 [287·79, 782·94] | < 0·001 | < 0·001 | < 0·001 | < 0·001 | < 0·001 |
| PD-L1, pg/mL | 263·55 [147·78, 446·83] | 280·83 [125·12, 584·67] | 0·87 | 0·64 | 0·85 | 0·88 | 0·93 |
| PCT, ng/mL | 4·24 [1·84, 6·09] | 0·58 [0·27, 2·71] | < 0·001 | < 0·001 | < 0·001 | < 0·001 | < 0·001 |
| CRP, pg/mL | 182·46 [91·46, 300·05] | 68·01 [25·21, 136·60] | < 0·001 | < 0·001 | < 0·001 | < 0·001 | < 0·001 |
| **Cytokine response** |  |  |  |  |  |  |  |
| IL-1β, pg/mL | 35·40 [22·80, 55·70] | 23·46 [14·45, 36·73] | < 0·001 | < 0·001 | < 0·001 | < 0·001 | < 0·001 |
| IL-6, pg/mL | 158·70 [50·18, 1166·93] | 48·08 [19·35, 147·98] | < 0·001 | < 0·001 | < 0·001 | < 0·001 | < 0·001 |
| IL-7, pg/mL | 9·45 [6·93, 13·26] | 8·29 [5·65, 10·88] | < 0·001 | < 0·001 | < 0·001 | < 0·001 | < 0·001 |
| IL-10, pg/mL | 6388·76 [506·90, 297951·32] | 47477·41 [1462·59, 1728617·08] | 0·057 | 0·047 | 0·061 | 0·053 | 0·12 |
| TNF, pg/mL | 64·64 [31·36, 127·22] | 25·16 [16·07, 50·56] | < 0·001 | < 0·001 | < 0·001 | < 0·001 | < 0·001 |
| GM-CSF, pg/mL | 255·72 [104·26, 836·83] | 584·64 [155·30, 1373·06] | < 0·001 | < 0·001 | < 0·001 | < 0·001 | < 0·001 |
| IFNγ, pg/mL | 9·01 [5·09, 18·71] | 10·65 [5·23, 25·49] | 0·67 | 0·65 | 0·70 | 0·72 | 0·70 |
| **Chemokine response** |  |  |  |  |  |  |  |
| CCL5, pg/mL | 4862·42 [3142·41, 8214·96] | 6788·57 [4225·19, 12622·73] | 0·003 | < 0·001 | 0·005 | 0·009 | 0·001 |
| CXCL8, pg/mL | 67·26 [28·76, 190·79] | 50·95 [23·39, 88·79] | 0·004 | 0·017 | 0·004 | 0·010 | 0·002 |
| CXCL10, pg/mL | 239·26 [90·29, 831·27] | 700·61 [220·92, 1058·29] | < 0·001 | < 0·001 | < 0·001 | < 0·001 | < 0·001 |

Depicted biomarker concentrations per group are for the main analyses. All data is presented as median [interquartile range]. Abbreviations can be found in the legend of Table S2. The main analysis includes all patients with a bacterial or a viral infection. Sensitivity analysis 1 excluded all patients with steroid treatment prior to sampling. Sensitivity analysis 2 excluded all patient with malignancies or auto-immune disorders. Sensitivity analysis 3 excluded all patients who received dialysis prior to sampling. Sensitivity analysis 4 excluded who received antibiotics for more than 24 hours prior to sampling. Statistical significance was calculated using a Welsh t-test with Benjamini-Hochberg correction for multiple testing. P-values are marked is the significance changes compared to the main analysis.

# Table S7. Comparison of biomarker concentrations between bacterial aetiologies unadjusted and adjusted for age, sex, and severity: main and four sensitivity analyses

|  | **Gram-positive bacteria** | **Gram-negative bacteria** | ***Leptospira sp.*** | ***O. tsutsugamushi*** | **Main** | **Sensitivity Analysis 1** | **Sensitivity Analysis 2** | **Sensitivity Analysis 3** | **Sensitivity Analysis 4** |
| --- | --- | --- | --- | --- | --- | --- | --- | --- | --- |
|  | **n = 56** | **n = 129** | **n = 72** | **n = 27** |  |  |  |  |  |
|  | **n = 52** | **n = 120** | **n = 64** | **n = 26** |  |  |  |  |  |
|  | **n = 52** | **n = 124** | **n = 72** | **n = 27** |  |  |  |  |  |
|  | **n = 54** | **n = 118** | **n = 68** | **n = 26** |  |  |  |  |  |
|  | **n = 50** | **n = 98** | **n = 64** | **n = 24** |  |  |  |  |  |
| **Endothelial and coagulation activation** | | | |  |  |  |  |  |  |
| ANG-1, pg/mL | 1780·44 [904·16, 4333·99] | 1242·07 [773·38, 2593·07] | 1205·92 [695·64, 1896·38] | 1626·61 [915·22, 2868·68] |  |  |  |  |  |
| ANG-2, pg/mL | 16500·19 [9621·61, 26901·34] | 22521·85 [8426·63, 42271·62] | 17515·70 [9729·11, 32280·61] | 17667·39 [7867·86, 29286·92] |  |  |  |  |  |
| ANG2/ANG1 ratio | 10·53 [3·36, 28·25] | 17·83 [3·98, 44·45] | 14·33 [7·02, 33·79] | 9·61 [4·31, 24·91] |  |  |  |  |  |
| Thrombomodulin, pg/mL | 11154·65 [8266·70, 17680·00] | 13638·98 [10107·13, 19219·31] | 12962·21 [9651·46, 17872·45] | 13054·98 [10211·69, 20997·10] |  |  |  |  |  |
| VCAM-1, pg/mL | 2478065·25 [1573443·30, 3751603·88] | 3504626·80 [1678584·87, 5294445·26] | 3064647·50 [1985851·85, 4795267·40] | 5717335·11 [3462959·59, 7326907·53] | A C c e f | A C c f | A C c e f | A C c e f | A c |
| Syndecan-1, pg/mL | 14015·22 [8088·51, 21562·65] | 16452·69 [9930·36, 26633·11] | 18107·99 [10288·68, 30448·59] | 26821·66 [21199·51, 39415·49] | A c e f | A c e f | A c e f | A c e f | A c e |
| D-dimer, pg/mL | 21929377·70 [12872952·17, 49547020·34] | 26531711·12 [12095786·76, 54671575·92] | 22065502·85 [13109986·86, 36304289·75] | 42290839·88 [17318886·98, 70529483·41] |  |  |  |  |  |
| Protein C, pg/mL | 78561·20 [52162·64, 112024·40] | 76594·71 [56487·77, 100994·94] | 83270·04 [55201·11, 103876·32] | 69669·95 [57919·76, 99504·43] |  |  |  |  |  |
| Platelets, 10^3/mL | 132·00 [85·25, 248·75] | 128·50 [70·00, 243·00] | 44·50 [26·50, 116·00] | 81·00 [43·50, 104·25] | A C b c d e | A C b c d e | A C b c d e | A C b c d e | A C b c d e |
| PT, sec | 15·05 [12·28, 24·60] | 15·00 [12·10, 21·90] | 11·45 [10·80, 15·14] | 14·10 [11·65, 15·95] | A C b d f | A C b d f | A C b d f | A C b d f | A C b d f |
| APTT, sec | 33·35 [28·05, 41·07] | 34·00 [27·60, 41·30] | 30·97 [27·42, 35·22] | 42·10 [31·60, 50·25] | A C f | C | A C f | A C c f | A C c e f |
| **Inflammation and organ damage** | | |  |  |  |  |  |  |  |
| RAGE, pg/mL | 4478·40 [2743·98, 6868·72] | 5849·93 [3342·29, 11154·09] | 5946·54 [2814·76, 9390·45] | 6997·07 [4183·41, 18339·42] |  |  |  |  |  |
| Ferritin, ng/mL | 561·43 [336·26, 1224·43] | 573·02 [274·03, 1174·67] | 380·98 [195·66, 783·31] | 1016·76 [683·20, 3427·41] | A C c e f | A C c e f | A C c e f | A C c e f | A C c e f |
| TNF RI, pg/mL | 1943·50 [1405·27, 2656·45] | 2417·04 [1693·22, 3480·59] | 2164·48 [1331·82, 3182·79] | 2239·90 [1430·51, 2868·28] | C | C |  |  |  |
| TREM 1, pg/mL | 886·52 [634·71, 1330·81] | 1038·37 [683·14, 1960·50] | 845·57 [599·93, 1460·49] | 706·21 [432·86, 1326·39] | C | A C | C | C | C |
| PD-L1, pg/mL | 236·40 [130·98, 365·77] | 243·04 [136·70, 401·42] | 290·60 [150·00, 482·45] | 635·75 [308·64, 1035·34] | A C c e f | A C c e f | A C c e f | A C c e f | A C c e f |
| PCT, ng/mL | 3·66 [1·95, 5·26] | 4·75 [2·87, 6·65] | 4·90 [2·10, 6·23] | 1·96 [1·18, 3·68] | A C c e f | A C c e | A C c e f | A C c e f | A C c e f |
| CRP, pg/mL | 216·33 [119·90, 321·85] | 213·91 [104·07, 327·60] | 183·97 [83·18, 264·52] | 195·23 [139·91, 297·34] |  |  |  |  |  |
| **Cytokine response** | |  |  |  |  |  |  |  |  |
| IL-1β, pg/mL | 34·61 [22·67, 55·49] | 38·27 [22·80, 60·22] | 33·22 [22·60, 48·62] | 39·74 [29·18, 60·82] | C | C |  |  | C |
| IL-6, pg/mL | 369·17 [109·65, 2832·71] | 248·09 [66·49, 2442·68] | 70·62 [31·63, 322·65] | 78·26 [39·66, 266·97] | A C b c d e | A C b c d e | A C b c d | A C b d | A C b d |
| IL-7, pg/mL | 9·10 [6·92, 11·90] | 10·15 [6·94, 14·17] | 8·37 [6·53, 11·62] | 9·63 [7·55, 12·07] | C | C | C | C | C |
| IL-10, pg/mL | 8725·48 [226·07, 86135·49] | 2413·45 [403·20, 28457·42] | 16935·08 [992·35, 624093·80] | 1751857·55 [104721·49, 6025744·78] | A C c e | A c e | A C c e | A c e | A c e |
| TNF, pg/mL | 43·46 [28·25, 77·81] | 64·72 [35·56, 157·66] | 69·19 [27·86, 176·93] | 79·87 [55·43, 140·98] | A C a c | A a c | A a c | A a c | A a c |
| GM-CSF, pg/mL | 242·11 [92·41, 624·81] | 159·60 [84·95, 378·09] | 517·30 [184·82, 1081·23] | 1305·96 [884·36, 1786·32] | A C c d e f | A C c d e f | A C c d e f | A C b c d e f | A C c d e f |
| IFNγ, pg/mL | 7·07 [5·07, 18·66] | 8·01 [5·09, 13·34] | 8·23 [4·16, 14·66] | 43·59 [17·93, 204·55] | A C c e f | A C c e f | A C c e f | A C c e f | A C c e f |
| **Chemokine response** | | |  |  |  |  |  |  |  |
| CCL5, pg/mL | 5770·35 [3899·17, 12675·17] | 4862·42 [3040·01, 7513·99] | 4050·39 [2846·41, 6215·54] | 4774·32 [3487·57, 6188·19] | A C b | A C b | A b | A b | A C a b c |
| CXCL8, pg/mL | 56·68 [28·56, 173·38] | 72·97 [31·29, 191·18] | 53·70 [19·90, 171·09] | 70·23 [34·01, 130·55] | C | C |  |  | C |
| CXCL10, pg/mL | 243·68 [81·98, 714·12] | 133·39 [71·20, 375·14] | 479·07 [144·38, 1021·91] | 1022·72 [703·28, 1176·56] | A C c d e f | A C c d e f | A C c d e f | A C c d e f | A C c d e f |

Depicted biomarker concentrations per group are for the main analyses. All data is presented as median [interquartile range]. The main analysis includes all patients with a mono-microbial bacterial sepsis aetiology. Sensitivity analysis 1 excludes all patients with steroid treatment prior to sampling. Sensitivity analysis 2 excludes all patient with malignancies or auto-immune disorders. Sensitivity analysis 3 excludes all patients who received dialysis prior to sampling. Sensitivity analysis 4 excludes who received antibiotics for more than 24 hours prior to sampling. Statistical significance was calculated with Welsh ANOVA for the unadjusted analysis and White-adjusted ANCOVA to correct for age, sex, and severity. Benjamini-Hochberg was used in both analyses to adjust for multiple testing. Pairwise comparisons were made using Games-Howell for all biomarkers that were significant in the unadjusted ANOVA. Abbreviations: A: ANOVA significant; C: ANCOVA significant; a: Gram-positive bacteria vs Gram-negative bacteria significant; b: Gram-positive bacteria vs *Leptospira sp.* significant: c: Gram-positive bacteria vs *O. tsutsugamushi* significant; d: Gram-negative bacteria vs *Leptospira sp.* significant; e: Gram-negative bacteria vs *O. tsutsugamushi* significant: f: *Leptospira sp.* vs *O. tsutsugamushi* significant. Biomarker abbreviations can be found in the legend of Table S2.

# Table S8. Comparison of biomarker concentrations between viral aetiologies unadjusted and adjusted for age, sex, and severity: main and four sensitivity analyses

|  | **Influenza** | **SARS-CoV-2** | **Dengue virus** | **KFD virus** | **Main** | **Sensitivity Analysis 1** | **Sensitivity Analysis 2** | **Sensitivity Analysis 3** | **Sensitivity Analysis 4** |
| --- | --- | --- | --- | --- | --- | --- | --- | --- | --- |
|  | **n = 62** | **n = 38** | **n = 26** | **n = 11** |  |  |  |  |  |
|  | **n = 52** | **n = 31** | **n = 26** | **n = 11** |  |  |  |  |  |
|  | **n = 60** | **n = 37** | **n = 26** | **n = 11** |  |  |  |  |  |
|  | **n = 59** | **n = 38** | **n = 25** | **n = 11** |  |  |  |  |  |
|  | **n = 55** | **n = 33** | **n = 25** | **n = 10** |  |  |  |  |  |
| **Endothelial and coagulation activation** | | | |  |  |  |  |  |  |
| ANG-1, pg/mL | 1987·69 [1037·46, 3793·39] | 3194·58 [1682·32, 7530·54] | 929·84 [734·68, 1653·63] | 1605·45 [1145·96, 2823·30] | A C b d | A C b d | A C b d | A C b d | A C b d |
| ANG-2, pg/mL | 6953·54 [3552·41, 13299·50] | 6075·68 [3416·14, 9729·08] | 13886·13 [5025·82, 27352·23] | 6823·87 [3696·96, 18795·25] |  |  |  |  |  |
| ANG2/ANG1 ratio | 2·65 [1·15, 6·55] | 1·64 [0·73, 4·11] | 17·14 [3·69, 22·55] | 4·35 [1·93, 7·38] | A C b d | A C b d | A C b d | A C b d | A C b d |
| Thrombomodulin, pg/mL | 8903·21 [7366·98, 11685·06] | 8681·10 [7274·30, 13688·09] | 9996·14 [7428·40, 12645·57] | 10530·41 [9499·43, 16016·04] |  |  |  |  |  |
| VCAM-1, pg/mL | 3479736·42 [2195702·77, 6319940·91] | 2275686·99 [1365585·67, 3896936·47] | 5659074·88 [3115706·43, 7326907·53] | 6291754·95 [4203936·49, 7890429·57] | A d e | A C d e | A a d e | A a d e | A C a d e |
| Syndecan-1, pg/mL | 9817·61 [6339·43, 16121·11] | 10845·75 [6554·58, 18425·44] | 20883·64 [14244·21, 31388·46] | 22452·80 [10595·69, 25070·87] | A b d | A C b d | A b d | A C b d | A C b d |
| D-dimer, pg/mL | 13282863·80 [8968217·58, 22310395·01] | 16801670·09 [8248958·18, 24800507·05] | 10797092·98 [8387857·02, 19152404·60] | 13909488·98 [9517315·50, 18424373·18] |  |  |  |  |  |
| Protein C, pg/mL | 81050·78 [61626·26, 110681·10] | 80539·43 [61695·93, 108347·79] | 82755·97 [66141·51, 111164·48] | 120383·38 [89701·98, 131169·22] |  |  | C |  |  |
| Platelets, 10^3/mL | 159·50 [123·00, 219·62] | 213·50 [137·75, 296·12] | 36·50 [17·00, 86·25] | 75·00 [55·00, 114·00] | A C b c d e | A C b c d e | A C b c d e | A C b c d e f | A C b c d e f |
| PT, sec | 12·00 [10·80, 13·67] | 11·50 [10·80, 13·60] | 12·00 [11·00, 13·74] | 12·00 [11·85, 13·73] |  |  |  |  |  |
| APTT, sec | 30·20 [26·35, 36·67] | 32·10 [27·60, 36·02] | 34·75 [27·60, 48·70] | 36·40 [26·20, 44·80] |  |  |  |  |  |
| **Inflammation and organ damage** | | | |  |  |  |  |  |  |
| RAGE, pg/mL | 9682·34 [2761·83, 23265·09] | 5047·28 [2434·03, 8164·02] | 3037·68 [2297·16, 3899·19] | 2997·84 [2294·02, 5887·71] | A C b | A C b | A C a b | A C b | A b |
| Ferritin, ng/mL | 484·55 [166·76, 1137·68] | 607·47 [328·49, 1171·85] | 2218·82 [777·26, 7201·12] | 4011·27 [1157·72, 5444·66] | A C b c d | A C b c d | A C b c d | A C b c d | A C b d |
| TNF RI, pg/mL | 1125·01 [838·46, 1514·26] | 1249·15 [836·95, 2305·88] | 1022·09 [789·44, 1180·62] | 1014·85 [976·69, 1252·58] |  |  |  |  |  |
| TREM 1, pg/mL | 465·86 [329·18, 912·33] | 538·58 [403·92, 791·05] | 372·51 [206·10, 572·52] | 296·78 [244·96, 548·60] | C |  | C | C |  |
| PD-L1, pg/mL | 316·99 [137·20, 548·57] | 201·89 [93·38, 293·51] | 455·81 [206·69, 729·12] | 803·03 [286·32, 1306·05] | A d e | A d e | A d e | A d e | A d |
| PCT, ng/mL | 1·14 [0·28, 3·80] | 0·47 [0·26, 1·52] | 0·48 [0·26, 2·27] | 0·37 [0·22, 0·49] |  |  |  |  |  |
| CRP, pg/mL | 73·14 [30·99, 144·06] | 70·81 [36·04, 132·24] | 59·70 [17·80, 135·06] | 51·04 [14·81, 105·80] |  |  |  |  |  |
| **Cytokine response** | | |  |  |  |  |  |  |  |
| IL-1β, pg/mL | 28·08 [15·34, 39·58] | 21·80 [10·32, 33·42] | 23·11 [18·03, 33·00] | 20·16 [15·24, 28·68] |  |  |  |  |  |
| IL-6, pg/mL | 67·63 [30·40, 255·70] | 36·58 [18·68, 80·26] | 31·75 [12·19, 132·97] | 26·89 [18·55, 97·40] |  |  |  |  |  |
| IL-7, pg/mL | 9·14 [6·94, 10·89] | 8·01 [5·75, 10·98] | 6·91 [5·62, 8·91] | 8·15 [4·33, 11·52] |  |  |  |  |  |
| IL-10, pg/mL | 67232·95 [3804·16, 2301230·63] | 1604·84 [257·97, 42253·99] | 3111981·99 [300168·46, 40824296·06] | 317340·00 [36521·39, 4575021·08] | A C a d e | A C a d e | A C a d e | A C d e | A C a d e |
| TNF, pg/mL | 24·09 [17·89, 49·23] | 20·26 [13·75, 55·43] | 29·78 [19·78, 46·82] | 29·99 [19·30, 43·37] |  |  |  |  |  |
| GM-CSF, pg/mL | 668·86 [310·41, 1392·82] | 147·18 [71·46, 578·10] | 1062·11 [819·26, 1746·10] | 800·08 [683·43, 1523·62] | A C a d e | A C a d e | A C a d e | A C a d e | A C a d e |
| IFNγ, pg/mL | 13·14 [6·63, 32·35] | 7·27 [4·10, 11·43] | 10·45 [5·36, 22·50] | 14·17 [5·04, 83·76] |  |  |  |  |  |
| **Chemokine response** | | |  |  |  |  |  |  |  |
| CCL5, pg/mL | 6956·53 [4381·58, 11310·33] | 12894·36 [5975·70, 17012·90] | 4949·10 [3239·23, 6467·82] | 6577·50 [4731·37, 9376·60] |  |  |  |  |  |
| CXCL8, pg/mL | 67·84 [28·12, 156·00] | 43·47 [19·33, 62·24] | 45·58 [18·03, 87·05] | 57·51 [46·85, 82·25] | A C a |  | A C a | A C a | C |
| CXCL10, pg/mL | 778·77 [352·76, 1119·24] | 277·62 [69·65, 882·45] | 899·21 [693·56, 1022·72] | 787·37 [629·27, 1141·55] | A C a d e | A C a d e | A C a d e | A C a d e | A C a d |

Depicted biomarker concentrations per group are for the main analyses. All data is presented as median [interquartile range]. The main analysis includes all patients with mono-microbial Influenza, SARS-CoV-2, dengue and Kyasanur Forest Disease infections. Sensitivity analysis 1 excludes all patients with steroid treatment prior to sampling. Sensitivity analysis 2 excludes all patient with malignancies or auto-immune disorders. Sensitivity analysis 3 excludes all patients who received dialysis prior to sampling. Sensitivity analysis 4 excludes who received antibiotics for more than 24 hours prior to sampling. Statistical significance was calculated with Welsh ANOVA for the unadjusted analysis and White-adjusted ANCOVA to correct for age, sex, and severity. Benjamini-Hochberg was used in both analyses to adjust for multiple testing. Pairwise comparisons were made using Games-Howell for all biomarkers that were significant in the unadjusted ANOVA. Abbreviations: A: ANOVA significant; C: ANCOVA significant; a: Influenza vs SARS-CoV-2 significant; b: Influenza vs dengue significant: c: Influenza vs Kyasanur Forest Disease significant; d: SARS-CoV-2 vs dengue significant; e: SARS-CoV-2 vs Kyasanur Forest Disease significant: f: dengue vs Kyasanur Forest Disease significant. Biomarker abbreviations can be found in the legend of Table S2.

# Table S9. Comparison of biomarker concentrations between bacterial and viral aetiology corrected for age, sex, and severity: main and four sensitivity analyses

|  | **Main** | | **Sensitivity analysis 1** | | **Sensitivity analysis 2** | | **Sensitivity analysis 3** | | **Sensitivity analysis 4** | |
| --- | --- | --- | --- | --- | --- | --- | --- | --- | --- | --- |
|  | **Bacterial (n=338), Viral (n=146; reference)** | | **Bacterial (n=310), Viral (n=129; reference)** | | **Bacterial (n=328), Viral (n=142; reference)** | | **Bacterial (n=318), Viral (n=140; reference)** | | **Bacterial (n=285), Viral (n=130; reference)** | |
| **Biomarkers** | **β (Confidence interval)** | **adj p-value** | **β (Confidence interval)** | **adj p-value** | **β (Confidence interval)** | **adj p-value** | **β (Confidence interval)** | **adj p-value** | **β (Confidence interval)** | **adj p-value** |
| **Endothelial and coagulation activation** | |  |  |  |  |  |  |  |  |  |
| ANG-1, pg/mL | -0·11 (-0·197 – -0·023) | 0·024 | -0·125 (-0·218 – -0·033) | 0·014 | -0·106 (-0·195 – -0·017) | 0·032 | -0·102 (-0·19 – -0·013) | 0·040 | -0·104 (-0·199 – -0·01) | 0·045 |
| ANG-2, pg/mL | 0·562 (0·407 – 0·717) | < 0·001 | 0·586 (0·419 – 0·752) | < 0·001 | 0·553 (0·395 – 0·71) | < 0·001 | 0·542 (0·383 – 0·701) | < 0·001 | 0·594 (0·425 – 0·763) | < 0·001 |
| ANG2/ANG1 ratio | 0·395 (0·273 – 0·517) | < 0·001 | 0·421 (0·29 – 0·552) | < 0·001 | 0·387 (0·262 – 0·511) | < 0·001 | 0·377 (0·252 – 0·503) | < 0·001 | 0·405 (0·27 – 0·539) | < 0·001 |
| Thrombomodulin, pg/mL | 0·151 (0·072 – 0·231) | < 0·001 | 0·166 (0·082 – 0·25) | < 0·001 | 0·164 (0·083 – 0·244) | < 0·001 | 0·149 (0·069 – 0·228) | < 0·001 | 0·16 (0·076 – 0·244) | < 0·001 |
| VCAM-1, pg/mL | -0·229 (-0·441 – -0·017) | 0·046 | -0·203 (-0·423 – 0·018) | 0·087 | -0·235 (-0·449 – -0·021) | 0·043 | -0·255 (-0·474 – -0·036) | 0·039 | -0·199 (-0·42 – 0·022) | 0·102 |
| Syndecan-1, pg/mL | 0·556 (0·041 – 1·071) | 0·046 | 0·589 (0·046 – 1·133) | 0·045 | 0·62 (0·1 – 1·141) | 0·032 | 0·522 (0·001 – 1·043) | 0·066 | 0·49 (-0·058 – 1·039) | 0·102 |
| D-dimer, pg/mL | 0·063 (0·028 – 0·098) | 0·001 | 0·076 (0·04 – 0·112) | < 0·001 | 0·065 (0·03 – 0·101) | < 0·001 | 0·059 (0·023 – 0·095) | 0·003 | 0·069 (0·031 – 0·107) | < 0·001 |
| Protein C, pg/mL | -5·535 (-10·184 – -0·887) | 0·032 | -5·363 (-10·255 – -0·472) | 0·045 | -5·168 (-9·904 – -0·432) | 0·043 | -4·985 (-9·787 – -0·183) | 0·062 | -6·636 (-11·489 – -1·782) | 0·013 |
| Platelets, 10^3/mL | 0·032 (-0·965 – 1·029) | 0·950 | 0·006 (-1·03 – 1·041) | 0·992 | 0·068 (-0·949 – 1·085) | 0·896 | 0·077 (-0·961 – 1·115) | 0·889 | 0·079 (-0·995 – 1·152) | 0·886 |
| PT, sec | 0 (0 – 0) | 0·038 | 0 (0 – 0) | 0·045 | 0 (0 – 0) | 0·043 | 0 (0 – 0) | 0·108 | 0 (0 – 0·001) | 0·021 |
| APTT, sec | 0·001 (-0·002 – 0·003) | 0·676 | 0·001 (-0·002 – 0·003) | 0·537 | 0·001 (-0·002 – 0·003) | 0·648 | 0 (-0·003 – 0·002) | 0·889 | 0·001 (-0·002 – 0·003) | 0·641 |
| **Inflammation and organ damage** | |  |  |  |  |  |  |  |  |  |
| RAGE, pg/mL | -0·012 (-0·029 – 0·005) | 0·189 | -0·008 (-0·026 – 0·01) | 0·437 | -0·013 (-0·03 – 0·005) | 0·183 | -0·015 (-0·032 – 0·002) | 0·113 | -0·011 (-0·03 – 0·007) | 0·268 |
| Ferritin, ng/mL | -0·098 (-0·161 – -0·034) | 0·005 | -0·102 (-0·169 – -0·035) | 0·005 | -0·1 (-0·164 – -0·036) | 0·004 | -0·088 (-0·153 – -0·022) | 0·016 | -0·105 (-0·174 – -0·036) | 0·006 |
| TNF RI, pg/mL | 0·486 (0·38 – 0·592) | < 0·001 | 0·523 (0·411 – 0·636) | < 0·001 | 0·495 (0·387 – 0·603) | < 0·001 | 0·485 (0·379 – 0·591) | < 0·001 | 0·5 (0·388 – 0·612) | < 0·001 |
| TREM 1, pg/mL | 0·215 (0·15 – 0·281) | < 0·001 | 0·242 (0·173 – 0·311) | < 0·001 | 0·217 (0·151 – 0·284) | < 0·001 | 0·21 (0·144 – 0·276) | < 0·001 | 0·229 (0·159 – 0·299) | < 0·001 |
| PD-L1, pg/mL | -0·08 (-0·157 – -0·003) | 0·051 | -0·088 (-0·169 – -0·007) | 0·045 | -0·08 (-0·158 – -0·002) | 0·058 | -0·079 (-0·158 – 0) | 0·066 | -0·064 (-0·146 – 0·017) | 0·147 |
| PCT, ng/mL | 1·269 (0·977 – 1·561) | < 0·001 | 1·338 (1·031 – 1·646) | < 0·001 | 1·256 (0·957 – 1·555) | < 0·001 | 1·281 (0·977 – 1·586) | < 0·001 | 1·306 (0·989 – 1·623) | < 0·001 |
| CRP, pg/mL | 5·292 (4·077 – 6·507) | < 0·001 | 5·057 (3·765 – 6·349) | < 0·001 | 5·301 (4·068 – 6·533) | < 0·001 | 5·441 (4·183 – 6·699) | < 0·001 | 5·252 (3·93 – 6·574) | < 0·001 |
| **Cytokine response** | |  |  |  |  |  |  |  |  |  |
| IL-1β, pg/mL | 0·342 (0·176 – 0·508) | < 0·001 | 0·399 (0·223 – 0·575) | < 0·001 | 0·354 (0·187 – 0·52) | < 0·001 | 0·36 (0·188 – 0·531) | < 0·001 | 0·357 (0·187 – 0·528) | < 0·001 |
| IL-6, pg/mL | 0·46 (0·294 – 0·627) | < 0·001 | 0·429 (0·253 – 0·606) | < 0·001 | 0·447 (0·278 – 0·616) | < 0·001 | 0·43 (0·26 – 0·601) | < 0·001 | 0·498 (0·318 – 0·678) | < 0·001 |
| IL-7, pg/mL | 0·137 (0·013 – 0·26) | 0·044 | 0·151 (0·02 – 0·283) | 0·037 | 0·139 (0·014 – 0·265) | 0·043 | 0·146 (0·018 – 0·274) | 0·040 | 0·163 (0·03 – 0·296) | 0·026 |
| IL-10, pg/mL | -0·716 (-1·121 – -0·312) | 0·001 | -0·709 (-1·137 – -0·282) | 0·002 | -0·722 (-1·134 – -0·309) | 0·001 | -0·757 (-1·174 – -0·34) | < 0·001 | -0·653 (-1·097 – -0·21) | 0·007 |
| TNF, pg/mL | 0·274 (0·199 – 0·349) | < 0·001 | 0·274 (0·195 – 0·352) | < 0·001 | 0·277 (0·201 – 0·354) | < 0·001 | 0·289 (0·211 – 0·366) | < 0·001 | 0·297 (0·217 – 0·378) | < 0·001 |
| GM-CSF, pg/mL | -0·603 (-0·821 – -0·386) | < 0·001 | -0·613 (-0·842 – -0·383) | < 0·001 | -0·611 (-0·832 – -0·39) | < 0·001 | -0·596 (-0·82 – -0·372) | < 0·001 | -0·577 (-0·812 – -0·341) | < 0·001 |
| IFNγ, pg/mL | -0·056 (-0·194 – 0·083) | 0·480 | -0·057 (-0·203 – 0·089) | 0·494 | -0·049 (-0·189 – 0·091) | 0·530 | -0·039 (-0·182 – 0·103) | 0·658 | -0·048 (-0·197 – 0·101) | 0·571 |
| **Chemokine response** | |  |  |  |  |  |  |  |  |  |
| CCL5, pg/mL | -0·339 (-0·684 – 0·006) | 0·066 | -0·451 (-0·801 – -0·101) | 0·019 | -0·326 (-0·68 – 0·028) | 0·086 | -0·291 (-0·639 – 0·056) | 0·117 | -0·402 (-0·768 – -0·035) | 0·045 |
| CXCL8, pg/mL | 0·046 (-0·076 – 0·168) | 0·495 | 0·029 (-0·099 – 0·157) | 0·683 | 0·049 (-0·075 – 0·173) | 0·492 | 0·032 (-0·093 – 0·157) | 0·668 | 0·068 (-0·062 – 0·197) | 0·343 |
| CXCL10, pg/mL | -0·459 (-0·598 – -0·32) | < 0·001 | -0·465 (-0·61 – -0·319) | < 0·001 | -0·459 (-0·601 – -0·317) | < 0·001 | -0·461 (-0·606 – -0·316) | < 0·001 | -0·437 (-0·589 – -0·285) | < 0·001 |

Association between infection etiology and biomarker levels corrected for age, sex, and severity (SOFA). Results are presented as β coefficients (B) with 95% confidence intervals (CI) derived from multivariable linear regression models comparing patients with bacterial infection to those with viral infection (viral infection as the reference category). The main analysis included all patients with either a bacterial or viral infection. Sensitivity analysis 1 excludes all patients with steroid treatment prior to sampling. Sensitivity analysis 2 excludes all patient with malignancies or auto-immune disorders. Sensitivity analysis 3 excludes all patients who received dialysis prior to sampling. Sensitivity analysis 4 excludes who received antibiotics for more than 24 hours prior to sampling. Benjamini-Hochberg was used to adjust for multiple testing.

# Supplementary figures

**
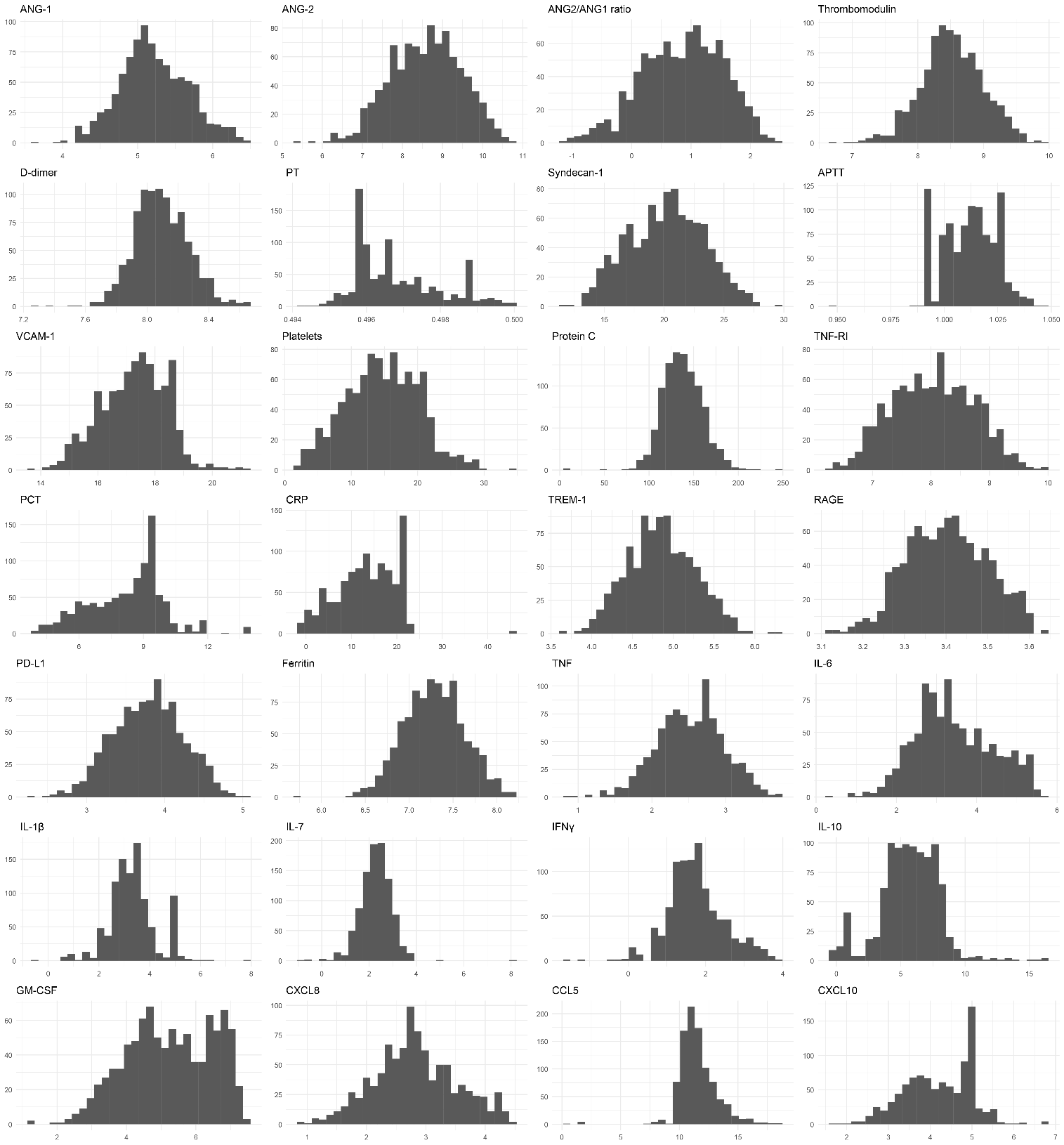
**

# **Supplemental Figure 1. Biomarker distribution plots after Box-Cox transformation**

Histograms showing the distribution of each biomarker after Box-Cox transformation.

**
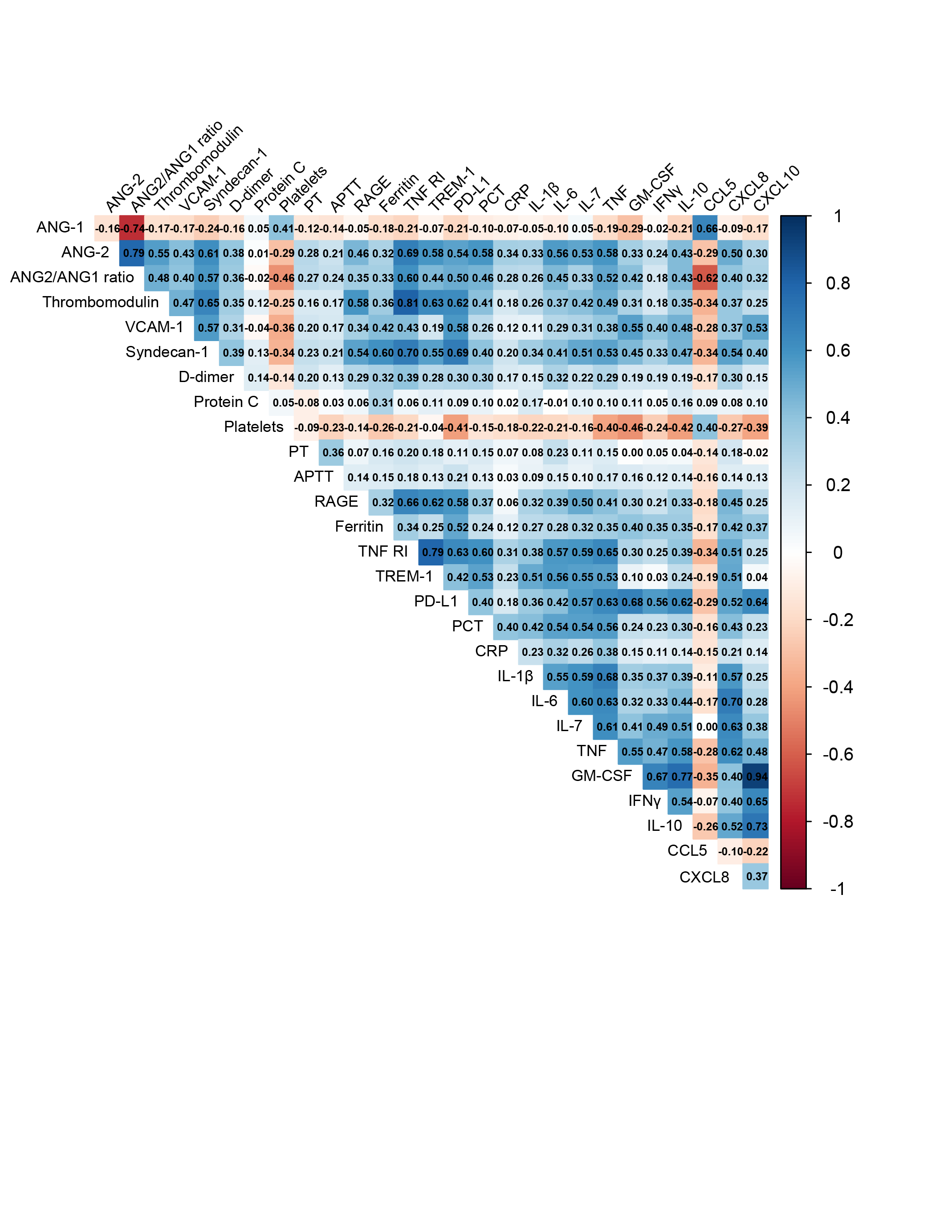
**

# Supplementary Figure 2. Correlation matrix of the scaled data underlying the PCA analyses.

Values represent pairwise correlations between biomarkers. Blue indicates positive correlations and red indicates negative correlations. The numerical values within the matrix denote the corresponding correlation coefficients.

**
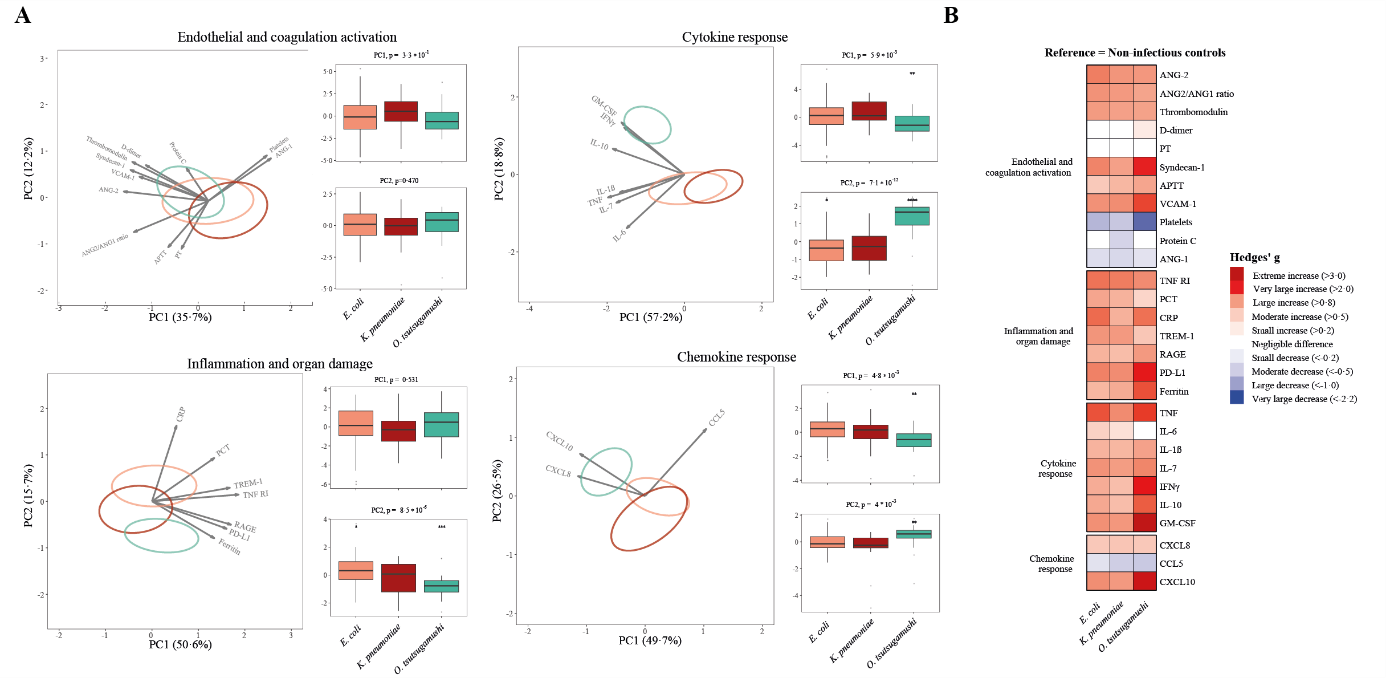
**

# **Figure S3. Comparison of host response profiles induced by O. tsutsugamushi with those induced by the two most common “classical” pathogens (E. coli and K. pneumoniae).**

A) Principal component analysis (PCA) of biomarkers per pathophysiological domain. Principal components (PC) 1 and 2 are plotted per domain. X-axis label shows the percentage of variance explained by PC1, Y-axis label shows the percentage of variance explained by PC2. The ellipse illustrate the area containing the most central 10% of patients for each group, centred on the group mean position. The arrows indicate the direction (arrow orientation) and strength (arrow length) of the correlation between each biomarker and the PCs. Box plots display PC1 and PC2 distributions. B) Heatmap depicting the Hedges’ g between the disease groups across pathophysiological domains with non-infectious ICU controls as reference. For abbreviations see legend of Table S1.
